# Economic and labour market impacts of migration in Austria: an agent-based modelling approach

**DOI:** 10.1186/s40878-024-00374-3

**Published:** 2024-03-26

**Authors:** Sebastian Poledna, Nikita Strelkovskii, Alessandra Conte, Anne Goujon, Joanne Linnerooth-Bayer, Michele Catalano, Elena Rovenskaya

**Affiliations:** 1 IIASA, Schlossplatz 1, 2361 Laxenburg, Austria; 2 Joint Research Centre, European Commission, Ispra, Italy; 3 Faculty of Computational Mathematics and Cybernetics, Lomonosov Moscow State University, Moscow, Russia

**Keywords:** E27: forecasting and simulation: models and applications, F22 international migration, J11 demographic trends, Macroeconomic effects, Forecasts, J15 economics of minorities, Races, Indigenous peoples, Immigrants

## Abstract

This study examines the potential economic and labour market impacts of a hypothetical but plausible migration scenario of 250,000 new migrants inspired by Austria’s experience in 2015. Using the agent-based macroeconomic model developed by Poledna et al. (Eur Econ Rev, 151:104306, 2023. 10.1016/j.euroecorev.2022.104306, the study explores the detailed labour market outcomes for different groups in Austria’s population and the macroeconomic effects of the migration scenario. The analysis suggests that Austria’s economy and labour market have the potential to be resilient to the simulated migration influx. The results indicate a positive impact on GDP due to increased aggregate consumption and investment. The labour market experiences an increase in the unemployment rates of natives and previous migrants. In some industries, the increase in the unemployment rates is more significant, potentially indicating competition among different groups of migrants. This research provides insights for policymakers and stakeholders in Austria and other countries that may face the challenge of managing large-scale migration in the near future.

**Supplementary Information:**

The online version contains supplementary material available at 10.1186/s40878-024-00374-3.

## Introduction

Austria, as well as most other advanced economies, has repeatedly experienced large waves of migration (Fassmann & Münz, 1995). In recent history, several events have led to an increase in migration flows to Austria: the Gastarbeiter (guest workers) policy in the 1970s implemented to counteract the lack of workers, which led to migration from Turkey and Yugoslavia; the fall of the Iron Curtain, which steered an increase in migration from central European countries and Yugoslavia, along with the Bosnian war (Jestl et al., 2022; Muckenhuber et al., 2022; Rupnow, 2017). In 1995, when Austria became a member of the European Union, migration increased the share of EU citizens, especially from Germany (Goujon & Bauer, 2015). Among the recent migration waves, the most significant was in 2015, around the peak of the Syrian civil war, when 88,000 asylum seekers originating from countries of the Middle East, including Syria, Afghanistan, and Iraq, entered the country. This wave was particularly notable as it marked the first major migration from regions outside Europe into Austria. In 2022–2023, Austria has been experiencing another wave of migrants seeking temporary protection from the ongoing war in Ukraine, accompanied by a spike in asylum applications from Afghanistan, India, Tunisia, and Morocco (Jestl & Tverdostup, 2023).

According to the United Nations High Commissioner for Refugees (UNHCR), as of mid-2023, Austria hosted about 277,000 refugees and subsidiary protection holders and over 35,000 asylum seekers, mainly from Ukraine, Syria and Afghanistan (UNHCR, 2023). With increasing geopolitical instability and economic insecurity, as well as worsening climate-related disasters, it is likely that Austria and other European countries will experience more frequent and sizeable waves of migration from countries in Africa and the Middle East (Migali et al., 2021). Mayrhofer and Ammer (2022) found that applicants for asylum in Austria increasingly refer to natural disasters as reasons for leaving their home countries or for their inability or unwillingness to return. Additionally, the effects of natural disasters are progressively being considered in the legal rationale of decisions on international protection made by the Austrian appellate court (Mayrhofer & Ammer, 2022).

The extent to which immigration affects the labour market and the wider economy in the short, medium and long term is the subject of ongoing debate. In the short term, immigration can increase the demand for goods and services, resulting in an increase in production and, consequently, the demand for labour, which, in turn, may lead to an increase in investment over time (Peri, 2010). Long-term effects have been studied more extensively, with many studies finding positive effects on growth, wages and employment in advanced economies (Alesina et al., 2016; Docquier et al., 2014). Ortega and Peri (2014) generalize these results and find a positive impact of immigration on per capita income in a large sample of countries. The impact of immigration on Gross Domestic Product (GDP) per capita is also shown to be positive in the long and short term from an empirical point of view (d’Albis et al., 2018, 2019).

The long-run impact of immigration on the labour market has been found to be either positive or insignificant for natives, depending on the skills of the immigrant labour force (Brücker et al., 2014; Borjas, 2014; Edo & Toubal, 2015; Ottaviano & Peri, 2012). Research suggests that an inflow of low-skilled immigrants can undermine close substitutes (i.e., low-wage natives and recent immigrants), who are more vulnerable to labour supply shocks. If immigrants and natives are imperfect substitutes, e.g., if they have the same education level but differing linguistic skills, there is more upward occupational mobility for native workers, allowing them to move to more complex jobs with higher wages and skills (Card, 2009; Manacorda et al., 2012). Additionally, highly skilled immigrants can generate positive externalities and, in some cases, can even drive long-term economic growth (Peri, 2016).

In this paper, we model and analyse the short to mid-term impact of a hypothetical yet plausible migration scenario on the labour market and the wider economy of Austria. The objective of this analysis is to understand the resilience of the Austrian economy and the labour market to migration shocks. It aligns with the recent priorities of the Austrian government emphasising rapid integration of migrants into the labour market as a primary policy objective (Bešić & Ortlieb, 2023). We adopt a macroeconomic approach to investigate the influence of immigrants on the economy of the host society, aligning with the broader examination of labour markets in the presence of migrants (Martin, 2022). This study aligns with the efforts of some European scholars, exemplified by Kahanec and Zimmermann (2010), who focus on the macroeconomic and labour market consequences of immigration (Dustmann et al., 2017; Hollifield, 2020).

Our scenario focuses on a large (250,000 people) influx of migrants. It is inspired by the 2015 experience in Austria, and we model the migrants based on the characteristics of the asylum seekers who came at that time. However, we simplify in the sense that we assume that the hypothetical influx has similar access to the labour market in Austria as citizens of countries other than the EU/EFTA, i.e. being granted asylum in Austria immediately after immigration. The focus is on the detailed labour market outcomes for different cohorts of the Austrian population (employed, unemployed, inactive, or retired, differentiated by sex, citizenship and industry of occupation, and totalling over a thousand cohorts) and the resulting macroeconomic consequences of such a migration scenario.

We analyse the migration scenario through the lens of a macroeconomic agent-based model (ABM) (Poledna et al., 2023), which we adapt to explore the consequences of a significant influx of migrants into Austria. The model consists of: (i) a large number of interacting heterogeneous agents such as households, government, banks, and firms, (ii) agents who interact in markets through search and matching processes, and (iii) a set of behavioural rules that move away from the assumptions of perfect rationality and information completeness to rules based on behavioural heuristics and bounded rationality. To analyse the effects of the migration scenario, the model of Poledna et al. (2023) is augmented with a more sophisticated household sector and calibrated to match key Austrian economic indicators in 2019,^1^ such as GDP growth, inflation rate, unemployment rate and budget deficit, as well as other economic and demographic indicators.

Several unique characteristics of the model are crucial for our analysis. First, the model takes into account the heterogeneous characteristics of the Austrian population and simulates the effects of migration on more than a thousand cohorts differentiated by sex, citizenship, activity status (e.g., inactive, unemployed, employed), and occupation (industry). Second, the labour market outcome for each of the analyzed cohorts is driven by behavioural, institutional, and socio-economic factors, which ultimately determine the labour market demand-supply match for native and non-native populations. To capture such non-market factors, probabilities of employment for a particular cohort in each industry are estimated based on data. Third, the model also allows for feedback effects between labour market outcomes and the general state of the economy through the interaction of agents in the labour, consumption and capital markets in the model.

The remainder of the manuscript is structured as follows. The “Related literature” section provides a summary of the relevant literature. “The model” section discusses the main characteristics of the ABM. The “Model calibration and definition of scenarios” section describes the data used to inform the model and introduces the hypothetical but realistic migration scenario constructed for the analysis. The “Results” section examines the aggregate and distributional impacts of the migration scenario, focusing in particular on the labour market outcomes of each cohort. The “Discussion and conclusions” section discusses the limitations and policy implications of our study and concludes that a data-rich and large-scale macroeconomic ABM can provide detailed information on the economic impacts of a sizable migration shock and show that the effects of migration differ across sex, citizenship, activity status, and occupation (industry).

## Related literature

The economic consequences of immigration have been extensively discussed in the literature. Two recent publications, Edo et al. (2020) and Hennessey and Hagen-Zanker (2020), provide comprehensive reviews of the state-of-the-art literature on this topic. Along with aggregated macroeconomic effects, such as changes in GDP or GDP per capita, researchers have focused their attention on labour market effects, including changes in employment and wages, and fiscal effects, by comparing public spending on benefits paid to immigrants with their tax contributions. For all kinds of consequences, it is important to differentiate between the short, medium, and long-term (Edo et al., 2020).

From a macroeconomic perspective, the impact of migration on GDP per capita has been empirically shown to be positive in the short and long run for advanced economies (Alesina et al., 2016; d’Albis et al., 2018, 2019). Moreover, most of the structural and non-structural empirical studies come to a consensus that in the long term, the impact of migration on employment and wages for the native population is either negligible or slightly positive, as outlined in Edo et al. (2020). Imperfect substitutability between natives and immigrants leads to more positive long-term effects (Edo et al., 2020). At the same time, the evidence is mixed regarding the short-term effects of migration on employment and wages. For example, using a wage-setting approach, Brücker et al. (2014) show that wages and employment of both natives and already-existing migrants in Germany, Denmark and the UK were slightly negatively affected by immigrants. On the other hand, Friedberg (2001) finds no negative effects of immigration on the wages of natives in Israel. An extensive discussion of the reasons for such differences can be found in Dustmann et al. (2016) and Edo (2019).

A specific strand of non-structural empirical studies uses “natural experiments”, such as large and sudden migration waves often caused by political factors, to analyse both the short- and long-term effects of immigration on the labour market of receiving countries. Such studies tend to demonstrate an increase in unemployment among the natives and a decline in average wages in the short run, with a consequent rebound to (or even exceeding) the pre-migration levels (Edo et al., 2020). For example, Hunt (1992) and Edo (2020) study the impact of the large-scale migration from Algeria to France resulting from the 1962 Algerian War of Independence. Hunt (1992) identified an increase in the unemployment rate and a decline in the average wage among the French natives taking a time horizon of six years. However, Edo (2020) shows that the wages and unemployment rate had fully recovered by 1976.

Some studies also examine the distributional consequences of immigration on the labour market of the host country, i.e., by analyzing impacts on specific population subgroups, such as workers in selected occupations or previous cohorts of immigrants as noted by Cohen-Goldner and Paserman (2011), Dustmann et al. (2013), and Ottaviano and Peri (2012). The skill composition of immigrants and their substitutability with natives play an essential role in certain occupational groups with respect to the impact of immigration on employment and wages (Edo et al., 2020). The reason for immigration, whether for economic reasons, family reunification, or seeking asylum, is also essential for assessing its impact and migrants are typically differentiated by demographic and skill composition as well as their labour market accessibility.

Evidence on the fiscal impact of immigration on advanced economies is mixed, with differences attributed to the characteristics of migrants such as age, skill level, family situation and country of origin, and features of the host economies such as labour market structure, tax and welfare systems, and legal regimes governing migration (Hennessey & Hagen-Zanker, 2020; Christl et al., 2022). Various static accounting studies indicate either slightly positive or slightly negative fiscal effects of migration, overall converging to fiscal neutrality (Preston, 2014). Dynamic approaches, i.e., net present value and generational accounting, usually demonstrate a negative fiscal effect of migration in the short term but eventually become positive over time (Edo et al., 2020; Hennessey & Hagen-Zanker, 2020; Mayr, 2005). However, depending on the assumptions of the analysis, conclusions can vary greatly even for the same country and period, as noted by Hennessey and Hagen-Zanker (2020).

The employment prospects of refugees in Europe are less favourable than those of other migrants in the short-term with respect to both the employment rates and wages, improving, however, in the longer term (Ott, 2013; Brell et al., 2020). For Austria, a comprehensive analysis of the labour market integration of refugees and other (non-humanitarian) migrants in 2009–2018 conducted by Jestl et al. (2022) and Jestl and Tverdostup (2023) supports these conclusions. They found that both refugees and other migrants predominantly found their first jobs in low-wage segments and similar industries while refugees took longer to find their first job compared to other migrants. Educational level and sex were found to play a significant role in the labour market integration process, with women facing more challenges than men and higher-educated migrants experiencing slower job entry but better job quality. Regional labour market conditions also influenced job entry and stability, with more jobs found in urban areas. On the other hand, since the process of consideration of asylum applications is quite lengthy, and the overall number of positive asylum decisions (enabling the refugees to access the labour market) has been relatively small, the overall economic and labour market impacts of the 2015 refugee influx are assessed to be modest (Rengs et al., 2017). Rengs et al. (2017) also showed a general match between the labour supply of refugees who have arrived in Austria and the labour demand in the host economy suggesting a good labour market integration potential of recent refugees, particularly for those from Syria and Iraq.

From a methodological point of view, general equilibrium (GE) modelling enables the assessment of both direct and indirect (i.e., through the impact on wages, taxation, and interest rates) macroeconomic, labour market and fiscal effects of migration on receiving countries (Edo et al., 2020), typically over a long-term horizon (up to 80 years). GE models generally show that an influx of immigrants does not deteriorate the economic performance or fiscal balance of the receiving country. Moreover, GE models can identify certain characteristics of immigrants (such as age and skill level) that lead to positive macroeconomic consequences. For example, Holler and Schuster (2020) simulated the long-run (until 2060) economic effects of the 2015–2020 refugee influx to Austria using a dynamic full-scale numerical overlapping generations (OLG) model. The authors stratified the modelled population by age, skill level, origin (natives and refugees), and savings type. The model demonstrates the following long-term effects of the refugee influx scenario: (1) a positive impact on aggregate private consumption and GDP, (2) a reduction in per-capita GDP during the entire 40-year horizon, (3) a slightly negative impact on employment and wages of low-skilled workers, both native and immigrant, (4) negative short-run fiscal effects turning positive in the long run, and (5) higher public debt during the entire time horizon. This study also analyses the potential outcome of selected migration policies.

As an alternative approach to GE models, ABMs have been applied to assess macroeconomic, labour-market, and fiscal consequences of economic policy measures, such as monetary and fiscal policy, financial regulation, and structural reforms (see Dawid & Delli Gatti, (2018) for a comprehensive review). In contrast to GE models, macroeconomic ABMs have proven capable of modelling out-of-equilibrium economic dynamics, reproducing stylised facts, and accounting for bounded rationality on the part of individual and collective (e.g., firm) decision-makers. Furthermore, ABMs can distinguish short, medium and long-term effects of economic and other shocks and, importantly, address heterogeneity among economic agents, making it possible to study distributional effects and inequalities (Dawid and Delli Gatti, 2018).

ABMs also have the potential to overcome the limitations of traditional models in capturing the complexities of migration processes (Bijak, 2022). Bijak (2022) argues that ABMs can incorporate individual behaviours and interactions and allow for the simulation of various scenarios, including policy interventions, and can provide insights into potential outcomes of different migration policies, making them well-suited for exploring the dynamics of migration. ABMs have been applied to study various types of migration, from rural–urban (e.g., Silveira et al., 2006) to international migration. For a review, see Klabunde and Willekens (2016) and Oleynik et al. (2021).

However, such models usually focus on the causes and the processes of migration rather than on its impact on the destination countries, see, for example, Lin et al. (2016). These models simulate the decision-making systems of potential migrants and account for various relevant factors, such as availability of jobs and social benefits, social networks of compatriots in the destination country (Rehm, 2012), and openness of borders (Groen, 2016)—so-called “pull factors”, as well as safety of the current place of residence (Hébert et al., 2018) and satisfaction with the current job and income—so-called “push factors”, and availability of information along the migration routes (Hinsch and Bijak, 2023). For example, several studies incorporate the Theory of Planned Behavior (TPB) into ABMs to study the causes of decisions to migrate. Klabunde et al. (2017) extended a demographic multistate model by incorporating behavioural rules based on the TPB to explain transitions and intentions. The authors exemplified their approach by modelling migration from Senegal to France. Kniveton et al. (2011) employed TPB and different scenarios to simulate future migration flows in Burkina Faso and assess the impact of various factors on migration patterns. The decision-making process of each agent in this model is based on their experiences, biases, assets, and perceptions, which contribute to the heterogeneity of agents and their responses to environmental stimuli and the actions of others. There is a growing number of other ABMs analysing the impact of climatic and ecological conditions as drivers of migration, for example, Naivinit et al. (2010) and Janmaat et al. (2015).

Several recent studies use ABMs to analyse interactions between native and migrant populations and the impacts of migration on the host societies, such as assimilation and acculturation as well as segregation (Houy, 2019; Paolillo & Jager, 2020; Perez et al., 2019; Sahasranaman & Jeldtoft Jensen, 2019).

ABMs are also developed to support testing various policies aimed at controlling migration and its consequences for the host country. For example, using an automated ABM framework for predicting refugee movements developed by Suleimenova et al. (2017), Suleimenova and Groen (2020) investigated the effects of border closures and reduced camp capacity decisions on refugee arrivals in South Sudan. Simon et al. (2018) developed an ABM to evaluate how different visa restrictions might influence the flow of legal and unauthorized migration and Simon (2019) employed an ABM to study how network-driven migration from Mexico to the USA can respond to changes in immigration policy, especially when return migration is considered.

However, despite their potential advantages, ABMs have not yet been widely used to assess the economic consequences of immigration. To the best of the authors’ knowledge, only two publications have approached this topic. Kaufmann et al. (2018) developed a stylised ABM to study the effects of immigration on the labour and goods markets of a simple economic system in an equilibrium state. The simulations demonstrate a readjustment of the markets, which eventually results in a new equilibrium with both wages (in the labour market) and prices (in the goods market) decreasing proportionally. The wealth of agents remains almost unchanged. Makarov et al. (2022) analyse the competition between natives and immigrants for workplaces and the corresponding macroeconomic effects on an artificial economy. There are currently no studies that use an empirically calibrated and validated ABM to examine the economic impacts of selected immigration scenarios for a specific country.

## The model

In this study, we use the ABM developed by Poledna et al. (2023) and augment it with a more sophisticated labour market module. There are two main differences with respect to the model developed by Poledna et al. (2023). First, we differentiate individual agents according to their main labour-relevant attributes, including their sex, citizenship, activity status, and occupation (industry), which are calibrated based on census and labour market data (see “The agent-based model” section). Second, we develop a new labour market module involving a search-and-matching mechanism informed by transition probabilities. In this mechanism, the probability that an applicant is selected depends on the applicant’s labour-relevant attributes according to a calibrated labour market transition probabilities matrix. In “The agent-based model” section, we provide a brief overview of the ABM of Poledna et al. (2023) and focus on the new module of the labour market developed for this study in detail in “The labour market module” section.

### The agent-based model

The ABM of Poledna et al. (2023) includes individuals, firms, and government entities or agents. The firm agents are classified into financial and non-financial. For the latter group, the Statistical Classification of Economic Activities in the European Community (NACE) system classification applies (European Communities, 2008). Firm agents are further grouped into domestic and foreign firms. The domestic firm population of each industry is derived from business demography data, with heterogeneous firm sizes following a power law distribution, which approximately corresponds to the firm size distribution in Austria. Foreign firms consist of a single consolidated agent for each industry to account for trade with the rest of the world.

The household sector is populated by individual agents. Employed individuals supply labour to financial and non-financial domestic firms and earn wages. Unemployed individuals receive unemployment benefits; investors receive dividends as firm owners; inactive and retired individuals receive social benefits. All individuals purchase goods and services on the consumption market and can invest in housing. They follow a simple heuristic by consuming a fixed share of their income and deciding their investments based on expectations of future output and price levels.

Firm agents produce goods and services using labour, capital, and intermediate inputs that are produced by other firms according to a Leontief production function, which is calibrated based on data from input–output tables. To plan their activity, firm agents need to anticipate the future state of the economy, including future prices and demand. In this model, firms do so by estimating simple statistical models based on past data representing their experience.

Firms, households and government entities interact in the labour, credit, and goods/services markets according to a search-and-matching mechanism, whereby sellers (e.g., firm agents in the goods/services market or individual agents in the labour market) are matched with buyers. For matching, a randomised algorithm is used that accounts for trade frictions.

To expand their investment, production and consumption capacities, firms and households can take loans from the banking sector. Minimum collateral requirements constrain the loan supply. Agents who are net savers use deposit services provided by the banking sector. The interest rates on deposits and loans are set as a markup rule over the central bank policy rate. The monetary model is closed with a standard Taylor rule-setting policy rate to monitor excess inflationary pressure.

The government agent collects taxes to finance public spending and social benefits to the households and to pay interest on the public debt. The state budget deficit adds to the stock of public debt.

The full description of the ABM can be found in Poledna et al. (2023), where the model is discussed in detail, and the out-of-sample forecast performance of the model is evaluated. For the Austrian economy, it is shown that the model can compete with benchmark VAR and DSGE models in out-of-sample forecasting of macro variables. Description of the ABM version developed specifically for this study according to the ODD+D protocol, a widely used framework used to provide a structured and standardized way of describing ABMs (Müller et al., 2013), is provided in the Additional file 1.

### The labour market module

We retain the basic principles of the labour market module from Poledna et al. (2023), yet we expand the module by differentiating individual agents according to their main labour-relevant attributes. The activities of firm agents determine the demand for labour. Each firm uses labour as input for production and sets the target employment level according to the desired and expected level of economic activity and the average labour productivity. Employees can work part-time or overtime up to 150% of the standard full-time workload. Wages are prorated accordingly. Given the labour force in the previous time step, the expected required labour force in the next step, and taking advantage of the flexibility provided by part-time and overtime work, each firm sets a positive or negative number of vacancies. If negative, the firm dismisses individuals from its workforce randomly. Otherwise, if the demand for labour to reach the desired scale of activity is greater than the workforce in place, the firm posts labour vacancies, which represent the demand for new labour. Filling open vacancies occurs as an outcome of a simple search-and-matching mechanism informed by transition probabilities (see more details below).

The entire population of individual agents in the model consists of two age groups: 16–64 years old (working age) and 65+ years old (retired) individuals. We do not consider individuals below the legal working age (i.e., 15 years old) in the model. Individuals of working age are part of the labour market, while retired individuals are not. The activity status of working-age individual agents can be either economically active or economically inactive. Economically active individuals are either employed or unemployed and looking for a job. Economically inactive individuals are neither employed nor unemployed and include, among others, students. Economically active individuals are further grouped by the industry in which they work or have previously worked if they are unemployed. We use the NACE-level 2 classification of industries. It includes 88 industries; from these, we exclude 24 industries for which input–output tables are not available and two industries for which there are no meaningful input–output data. This leaves 62 industries.

Finally, in addition to sex, activity status and industry, individuals of working age are attributed with a citizenship status; we consider four states in the model: (i) Austrian citizens (referred to as “natives”), (ii) citizens of EU/EFTA countries other than Austria (referred to as “EU citizens”), (iii) citizens of countries other than EU/EFTA (referred to as “citizens of other countries”), and (iv) refugees. Technically refugees are citizens of countries other than Austria and EU/EFTA; they are placed into a separate sub-category (of individuals who have received “refugee status”) to analyse the effect of their joining the Austrian labour market. Table 1 summarizes the selected attributes and their values. Overall, the household sector consists of 1001^2^ cohorts of individuals.

The labour market facilitates dynamic transitions of individual agents to different activity statuses and industries mentioned above. For each sex- and citizenship-based cohort, transitions are (i) from inactive to active, i.e., an agent will search for a job across industries; (ii) from unemployed to employed in a given industry; (iii) from employed to employed in a different firm of the same or different industry; (iv) from employed to unemployed.^3^ An endogenous search-and-matching process conditioned by exogenously calibrated labour market transition probabilities governs transitions (i), (ii), and (iii). Individuals from all cohorts (except for retired individuals) apply to fill job vacancies. The probability that an applicant is selected by a firm from a given industry depends on the applicant’s cohort according to a labour market transition probabilities matrix. In our model, labour market transition probabilities $P_{ix}$ form a $62\times 1000$ matrix, where $x=1,\ldots , 1000$ is the cohort index specifying a combination of sex, activity status, citizenship and industry and $i=1,\ldots ,62$ is the industry in which a vacancy is open; $P_{ix}$ determines the probability that a random agent from cohort *x* is selected for an open vacancy in industry *i*. With data-informed heterogeneous labour market transition probabilities, the model can account for diverse social, institutional, or cultural factors that affect the likelihood that an individual is selected for a job in a particular industry without explicitly modelling complex decision processes at the firm level.

**Table 1:** Demographic attributes of agents-individuals and their values used in the model

Attribute	Sex	Citizenship	Activity status	Industry
Values		Natives	Employed	62 industries according to NACE-level 2 structure
	Men	EU	Unemployed	
	Women	Other countries	Inactive	
		Refugees	Retired	

## Model calibration and definition of scenarios

The ABM is calibrated to the Austrian economy using various data sources, including Eurostat, Statistics Austria, and the Database of Labour Market Information of the Austrian Federal Ministry of Labour and Economy. Austria is typical of an advanced small open economy with about 9.0 million inhabitants (in 2022) and more than half a million registered businesses. The calibration of the ABM to the Austrian economy is described in detail in Poledna et al. (2023). The time step of the model is one quarter of a year. In the “Calibration of the labour market module” section, we discuss the calibration of the labour market module developed for this study.

In the “Migration scenario” section, we develop a migration scenario, which is subsequently analyzed and compared with a baseline scenario of “no migration”. Since our purpose is to examine the resilience of the Austrian economy to migration, the scenario consists of a large migration wave; the scenario is hypothetical but plausible based on previous migration shocks, especially the 2015 migration wave, in terms of composition and subsequent integration of migrants in the Austrian labour market up until the current situation.

### Migration scenario

To analyze the resilience of and impact of immigration on the Austrian economy, two extreme, hypothetical scenarios are considered: (i) a baseline scenario and (ii) a migration scenario inspired by the 2015 experience in Austria. The baseline scenario assumes that there is no migration occurring throughout the entire simulation horizon. The migration scenario assumes sizable immigration of refugees over a period of six consecutive quarters, i.e., Q1–Q4 of year one and Q1–Q2 of year two. It also assumes that there are no economic or other migrants over this period.

**Fig. 1:**
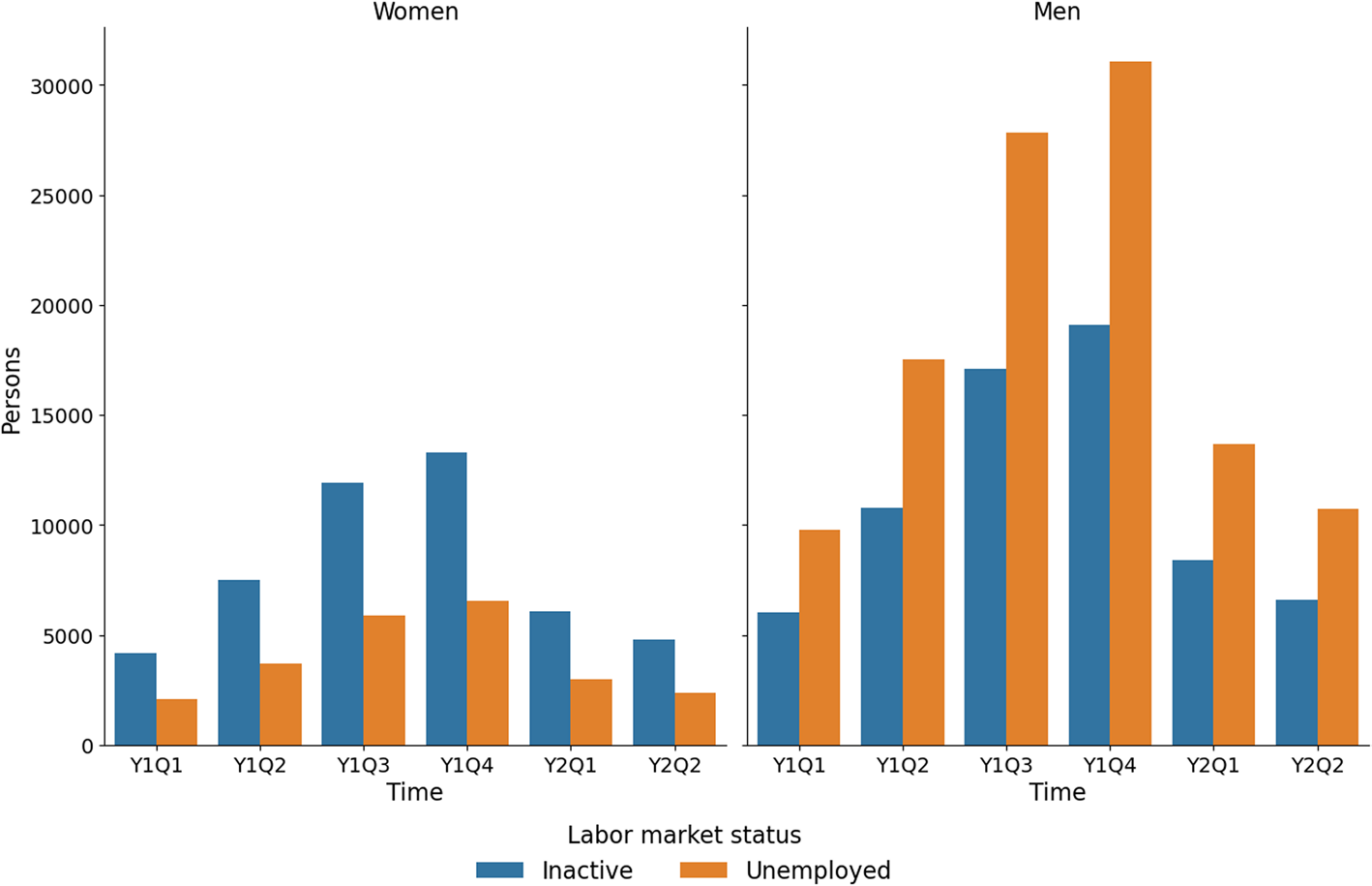
Number of arrivals over time in the migration scenario. Data source: own elaboration based on Eurostat (2023a); Buber-Ennser et al. (2016)

**Fig. 2:**
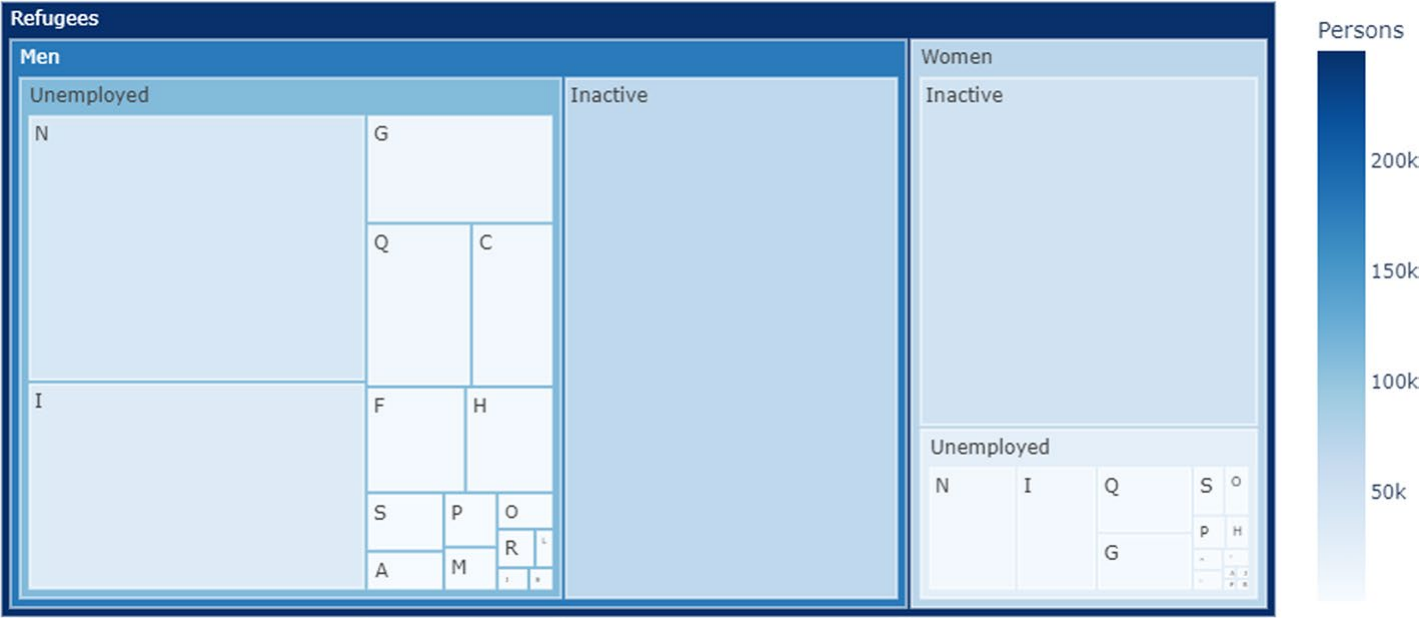
Composition of refugees in the migration scenario. The sizes of the rectangles denote the relative numbers of individuals in each cohort. For legibility reasons, industries in this figure are aggregated to NACE-level 1 structure (see Table 2); some industries are not presented in this figure as there are no individuals assigned to them in the data used. Data source: own elaboration based on Eurostat (2023a); Buber-Ennser et al. (2016); Bundesministerium für Arbeit und Wirtschaft (2023)

The parametrisation of the agents-refugees with respect to the individual attributes described in Table 1 in the migration scenario is derived from using several data sources as follows:

1. The total number of incoming refugees is assumed to be 250,000.^4^
2. For simplicity, we assume that the newly arrived migrants are granted asylum (i.e., the refugee status) in Austria immediately and have similar access to the labour market in Austria as citizens of countries other than the EU/EFTA (cf. Kaufmann et al. (2018)).^5^
3. The spread of arrivals over the six quarters and distribution of refugees by sex are similar to the refugee crisis of 2015–2016—in that case, most asylum applicants arrived in Austria from January 2015 to June 2016 (Eurostat, 2023a)—and represented in Fig. 1. There are more than twice as many men than women among refugees in our migration scenario. All arriving asylum seekers in our migration scenario belong to the working age.
4. The distribution of refugees for both sexes according to the activity status is assigned using the *Displaced Persons in Austria Survey (DiPAS)* conducted by the Wittgenstein Centre for Demography and Global Human Capital in 2015 (Buber-Ennser et al., 2016). Namely, data representing answers to Q25, “What do you plan to do in the future after you received a status (asylum or subsidiary protection)?” were used.
5. The distribution of unemployed refugees for both sexes across 62 industries is assigned using the data retrieved from the online Database of Labour Market Information (*Arbeitsmarktinformationssystem, amis*) maintained by the Austrian Federal Ministry of Labour and Economy (Bundesministerium für Arbeit und Wirtschaft, 2023), identical to the distribution of refugees already residing in Austria (see “Calibration of the labour market module” section)—see Fig. 2.
6. Refugees receive specific social transfers related to their refugee status of 560 Euros per month until they find employment.

**Table 2:** Structure of NACE-level 1, industries A–S (European Communities, 2008)

Industry	Title
A	Agriculture, forestry and fishing
B	Mining and quarrying
C	Manufacturing
D	Electricity, gas, steam and air conditioning supply
E	Water supply; sewerage, waste management and remediation activities
F	Construction
G	Wholesale and retail trade; repair of motor vehicles and motorcycles
H	Transportation and storage
I	Accommodation and food service activities
J	Information and communication
K	Financial and insurance activities
L	Real estate activities
M	Professional, scientific and technical activities
N	Administrative and support service activities
O	Public administration and defence; compulsory social security
P	Education
Q	Human health and social work activities
R	Arts, entertainment and recreation
S	Other service activities

### Calibration of the labour market module

Calibration of the extended labour market module of the ABM involves:

- Parametrization of the initial population of individual agents by setting the initial sizes of all defined individual agent cohorts based on their attributes of sex, citizenship, activity status, and industry, where applicable; and
- calibration of the labour market transition probabilities.

**Fig. 3:**
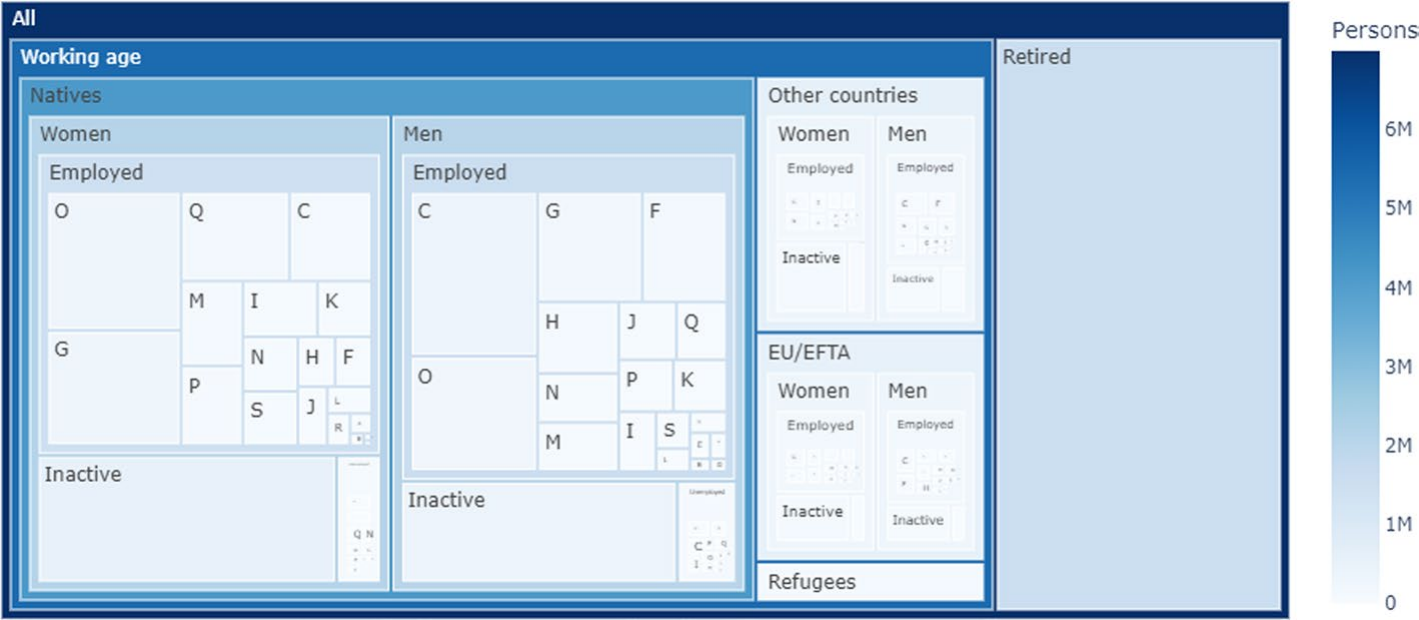
Composition of the initial population of individual agents in the model. The sizes of the rectangles denote the relative numbers of individuals in each cohort. For legibility reasons, industries in this figure are aggregated to NACE-level 1 structure (see Table 2). Data source: own elaboration based on Statistics Austria (2022b); UNHCR (2023); Bundesministerium für Arbeit und Wirtschaft (2023)

The initial size of all cohorts from Table 1, except for cohorts of refugee agents, utilizes the register-based census (*Registerzählung*) as reported by Statistics Austria (2022b). These data reflect all persons residing in Austria on a given date and their various attributes, including, among other attributes, their sex, activity status, citizenship and industry of occupation. We use data as of 31 October 2018 to obtain cohort sizes and initialize the model. The schematic representation of the initial population composition across cohorts of natives, EU citizens, and citizens of other countries is displayed in Fig. 3.

The register-based census data do not provide relevant data for refugees. To fill this data gap, we first distinguish refugee cohorts from cohorts of citizens of other countries. We obtained the number of refugees residing in Austria (in 2019) from the UNHCR database (UNHCR, 2023). Second, since neither Statistics Austria nor UNHCR provides data on the activity status and industry of occupation (where applicable) of refugees, we rely on data from the Online Database of Labour Market Information (Bundesministerium für Arbeit und Wirtschaft, 2023). We approximate the distribution of all refugees over sex, activity status and industry of occupation based on the corresponding distribution for refugees with Afghani, Iraqi, Somalian and Syrian citizenship, which together constitute more than 77% of refugees residing in Austria in 2019 (UNHCR, 2023).

The labour market transition probabilities for the over 1000 cohorts and 62 NACE-level 2 industries are estimated using the Register-based labour market careers statistics (*Registerbasierte Erwerbsverläufe; ERV*) provided by Statistics Austria (2022a). Namely, the probability that a job applicant will be accepted for employment in a given firm/industry is based on data on new employment across industries, as well as the number of individuals in each cohort (except for refugees), averaged over four quarters of 2019. Labour market transition probabilities for all cohorts of refugees are assumed to be equal to the labour market transition probabilities for the corresponding (in terms of sex, activity status and industry) cohorts of individuals from other countries.

**Fig. 4:**
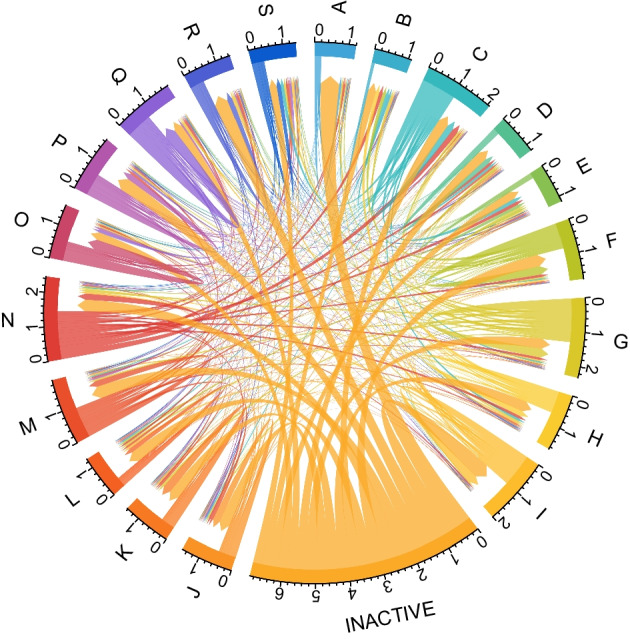
Labour market transition probabilities among different cohorts. The thickness of flows corresponds to the probability value. For legibility reasons, industries in this figure are aggregated to NACE-level 1 structure (see Table 2), employed and unemployed belonging to the same industry are aggregated, and both sexes are combined. Data source: own elaboration based on Statistics Austria (2022a)

Figure 4 visualises the estimated labour market transition probabilities. Notably, in many industries, the largest share of new employees was inactive before they were hired. The second largest share of new employees was working in the same industry before the transition (i.e., an individual was hired by another firm in the same industry).

## Results

In this section, we present the results of the model simulation in terms of the macroeconomic consequences of the migration scenario in comparison with the baseline (“Macroeconomic effects” section), as well as the impact on the labour market with the focus on the unemployment dynamics by industry and cohort (“Labour-market effects” section).

### Macroeconomic effects

**Fig. 5:**
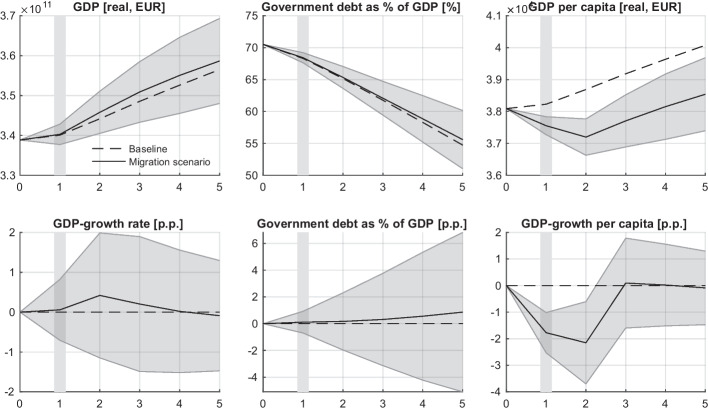
Macroeconomic indicators of the Austrian national economy in the baseline (broken line) and migration (solid line) scenarios over the simulation period of five years. The top panel shows GDP and per capita GDP in levels, as well as the government debt-to-GDP ratio in percentage. The bottom panel displays the differential impact of the migration scenario on the GDP growth rate and the per capita GDP growth rate, as well as the government debt-to-GDP ratio, expressed in percentage points (p.p.) relative to the baseline. The grey area around the migration scenario (solid line) provides an uncertainty range (one standard deviation)

Given the recent Austrian budget deficit, we assume that the additional government spending to finance expenses arising from the migration shock translates into an increased budget deficit or public debt. Direct extra expenses arise from social transfers to newly arrived refugees. Due to economy-stimulating multiplier effects resulting from extra consumption, and caused by it extra production, the resultant burden on the public debt can be lower. According to simulations, the additional public debt due to migration is approximately one percentage point (p.p.) of the GDP after five years in the migration scenario (the bottom mid panel in Fig. 5). This effect appears relatively marginal compared to the 15 p.p. of the overall decrease of the Austrian public debt relative to the GDP over the five years of simulation in the baseline scenario without migration. This decrease is due to the assumption of unchanged fiscal policy, which is used to focus on the effects of the migration scenario. In reality, a decrease in public debt might motivate the government to soften the fiscal policy, by, for example, introducing tax reductions. In absolute numbers, the additional government spending required to finance social transfers for new refugees over five years is approximately 7.4 billion Euros, which is about 2.7% of the Austrian public debt by the end of the simulation period. However, economic multiplier effects can result in a reduction of 2.4 bln. Euros or about 30% of the extra expenditures. Thus the resultant extra burden on the public debt is 1.1 p.p. as mentioned above (illustrated in Fig. 5).

The government spending has a positive impact on the national economy by creating a demand for goods and services among consumers and further positive indirect effects, including higher investment and labour demand. As a result, the baseline economic growth rate of 1.2% per year is expected to increase by about 0.5 p.p in year 2 and then return to the baseline rate in the course of the next three years of simulation.

As shown in Fig. 5, the Austrian per-capita GDP drops by about 2% compared to the baseline scenario in the first year after the arrival of 250,000 migrants (about 2.8% of the country population) and then converges to the baseline growth rate of 1.2% over the next two years. However, in absolute terms, the GDP per capita does not fully recover over the 5-year simulation period.

These results corroborate those of Holler and Schuster (2020), who found a positive impact of refugee migration to Austria in 2015 in terms of the aggregate consumption and the GDP accompanied by a reduction of the GDP per capita in the short- and long-run based on their overlapping generations (OLG) model of Auerbach-Kotlikoff type.

### Labour-market effects

**Fig. 6:**
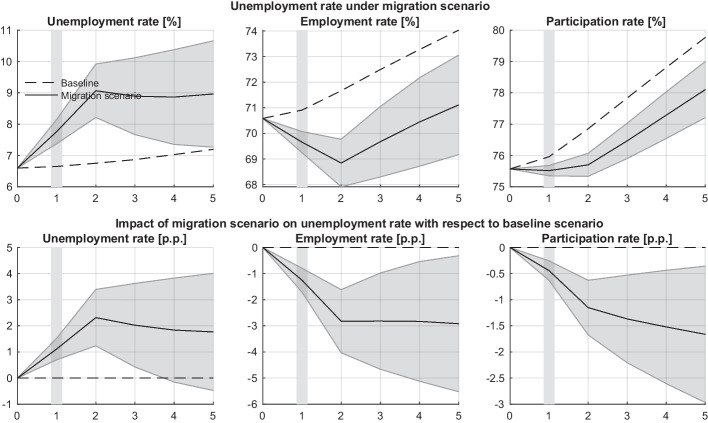
Population-wide unemployment, employment and participation rates in the baseline (broken line) and migration scenarios (solid line) over the simulation period of five years. The top panel shows overall unemployment, employment, and participation rates for both scenarios. The bottom panel displays the differential impact of the migration scenario on these rates, expressed in percentage points (p.p.) relative to the baseline. The grey area around the migration scenario (solid line) provides an uncertainty range (one standard deviation)

**Table 3:**
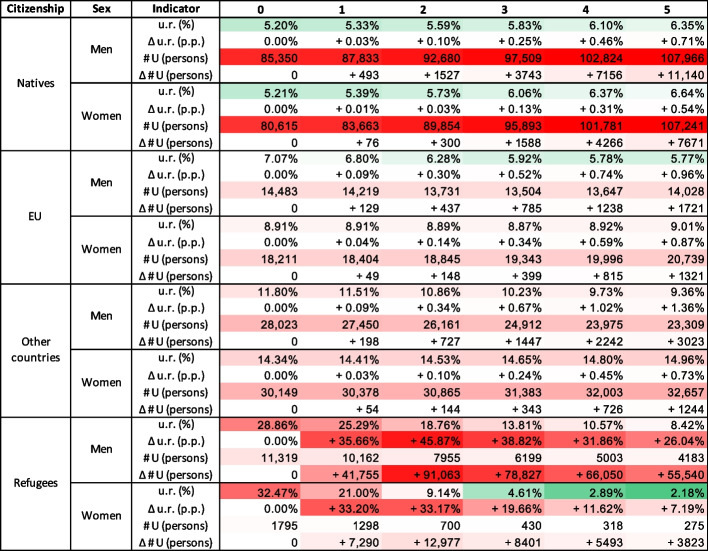
Disaggregated impacts of the migration scenario on the labour market by cohort

u.r.: Unemployment rate in the baseline scenario; $\Delta$ u.r.: Difference in the unemployment rate (in p.p.) between the migration scenario and the baseline scenario; $\# U$: Absolute number of unemployed persons in the baseline scenario; $\Delta \# U$: Difference in the absolute number of unemployed persons between the migration scenario and the baseline scenario

In the model’s baseline scenario, both the employment and participation rates^6^ are expected to increase over time, by about 0.8 p.p. and 1 p.p. per year, on average, respectively. The participation rate increases faster than the employment rate because, according to Austrian data, economically inactive people have, on average, a higher chance of becoming employed compared to their unemployed counterparts.^7^ As a result, the unemployment rate slightly increases from 6.6% in year 1 to 7.2% in year 5, for the same reason as discussed above.

When migrants enter the labour market, it experiences a positive supply shock and, as a direct effect, the unemployment rate increases from 6.6% to 9.1% in the second year and remains at that level until the end of the simulation period (Fig. 6, top panel). Similarly, the employment and participation rates decrease by about 3 p.p. and 1.1 p.p. in the second year, respectively (Fig. 6, bottom panel).

The model simulates the unemployment rates across a total of 496^8^ population cohorts. In Table 3, we present the model results disaggregated by sex and citizenship, in Table 4 by industries, and Table 5 by industry, sex, and citizenship for several selected industries.

Table 3 shows that within the cohorts of natives, EU citizens, and other country citizens, the migration shock leads to a disproportionally higher increase in unemployment among men compared to women. This effect is the most pronounced within the cohort of other country citizens, where the difference reaches approximately 0.63 p.p. in year 5, and is small within the cohort of natives (0.17 p.p. difference) and EU citizens (0.09 p.p. difference). Of these six cohorts, men - citizens of other countries are the most affected with a 1.36 p.p. increase in unemployment in year 5.

These observations should be contrasted with the fact that in the baseline scenario, unemployment among women is higher than among men across all three cohorts. Indeed, the unemployment gap between men and women is widening over time, with women remaining more likely to be unemployed than men.

Comparing across citizenship cohorts, we observe that natives are generally less affected than EU citizens, who, in turn, are less affected than citizens of other countries. As a result of the migration scenario, there are approximately four thousand additional unemployed individual agents in this cohort in year 5, which is about 0.69% of the total number of such persons. Despite a lower unemployment rate and a lower unemployment rate change, due to the large size of the cohort, there are a total of about 19 thousand additional unemployed natives in year 5, which constitutes 0.45% of this cohort size.

The unemployment rate among refugees shows an opposite pattern both temporally and according to sex in both the baseline and migration scenarios. In the baseline scenario, the unemployment rate among both men and women refugees is significantly decreasing over time, demonstrating success of integration of refugees into the Austrian labour market. It’s worth noting that in the baseline scenario, no new refugees arrive during the entire simulation period. Under this assumption, in the baseline scenario, the end-of-period unemployment rates among refugees are lower than among other countries citizens. The migration scenario brings about 35.66 p.p. and 33.20 p.p. increases in unemployment among men and women refugees in year 1, respectively. Over time, the extra unemployment decreases reaching 26.04 p.p. and 7.19 p.p. for men and women in year 5, respectively. In both scenarios, female refugees are significantly more successful in integrating into the Austrian labour market than their male counterparts. However, we note that the number of women refugees in our migration scenario is lower than men.

**Table 4:**
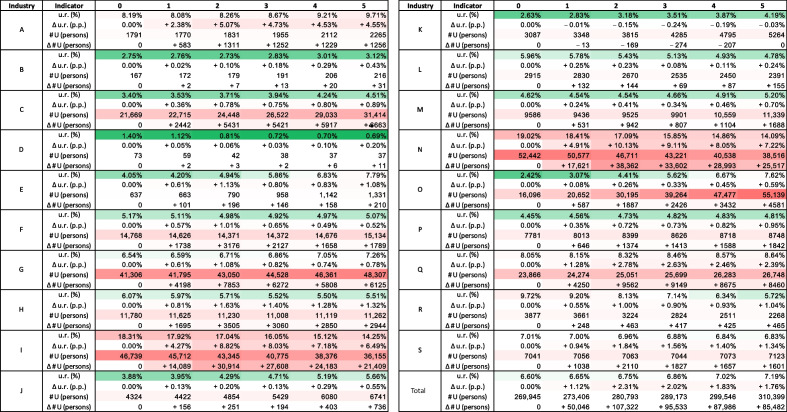
Disaggregated impacts of the migration scenario on the labour market by industry

u.r.: Unemployment rate in the baseline scenario; $\Delta$ u.r.: Difference in the unemployment rate (in p.p.) between the migration scenario and the baseline scenario; $\# U$: Absolute number of unemployed persons in the baseline scenario; $\Delta \# U$: Difference in the absolute number of unemployed persons between the migration scenario and the baseline scenario

**Fig. 7:**
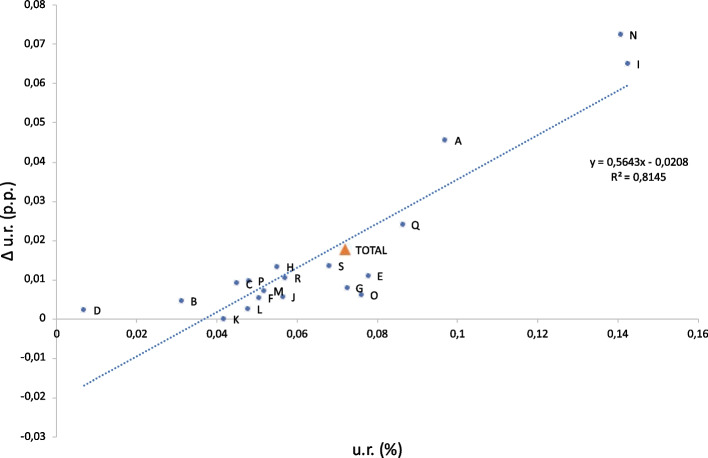
Unemployment rate change in the migration scenario depending on unemployment rates in the baseline scenario for 19 NACE-level 1 industries in year 5. The dotted blue line depicts a linear regression line between the two variables; the estimated slope is 0.56 with P-value=0.0000001

More insights can be obtained by disaggregating the impact of migration on the labour market into industry-specific unemployment rates, presented in Table 4. The impact in most industries is negative, consistent with aggregated results discussed above, but varies in magnitude. The range of effects across industries is as follows:

- *Administrative and Support Service Activities* (industry N) and *Accommodation and Food Service Activities *(industry I) experience the highest negative impacts (+7.22 p.p. and +6.49 p.p. by year 5, respectively). These are industries, in which a substantial number of incoming refugees were specialized.
- They are followed by *Agriculture, Forestry and Fishing* (industry A) (+4.55 p.p.) and *Human Health and Social Work Activities* (industry Q) (+2.39 p.p.). Industry A received relatively few refugees but, in relative terms, appears to be strongly affected.
- Most other industries have effects of magnitude lower than 2 p.p. (and most lower than 1 p.p.).
- *Financial and insurance activities* (industry K) experiences a slight positive effect ($-$0.03 p.p.).
- Importantly, industries with already high unemployment generally experience higher unemployment increases than industries with low unemployment (see Fig. 7). This conclusion is independent of whether relative or absolute numbers are used to evaluate unemployment and shock-induced additional unemployment.
- Another important result is that there are a few industries that demonstrate the capacity to “absorb” more workers. For example, in *Electricity, Gas, Steam and Air Conditioning Supply* (industry D), *Real Estate Activities* (industry L), and *Arts, entertainment and recreation* (industry R), the unemployment rate decreases over the simulation period in the baseline scenario and even in the migration scenario by year 5, it is lower than the unemployment rate in year 0, that is, before refugees arrive, constituting 0.9%, 5%, and 6.8% respectively, which is also lower than the national unemployment rate of 9.1%. This pattern suggests that these industries might be in need of labour.

**Table 5:**
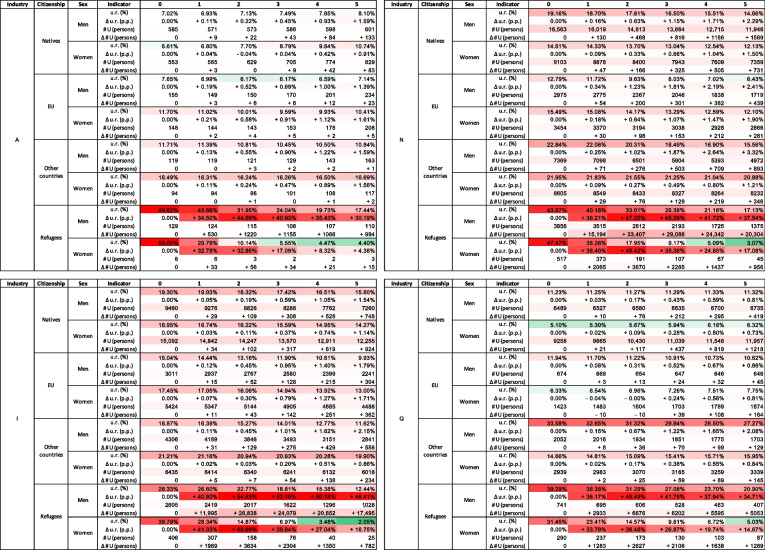
Disaggregated impacts of the migration scenario on the labour market for four most affected industries

u.r.: Unemployment rate in the baseline scenario; $\Delta$ u.r.: Difference in the unemployment rate (in p.p.) between the migration scenario and the baseline scenario; $\# U$: Absolute number of unemployed persons in the baseline scenario; $\Delta \# U$: Difference in the absolute number of unemployed persons between the migration scenario and the baseline scenario

The analysis presented earlier shows that the impact of the migration shock on unemployment varies significantly across sex, citizenship and industry cohorts. Table 5 presents the impacts disaggregated according to these attributes combined for the four industries most negatively affected by unemployment: industries N, I, A, and Q. As industry-specific unemployment rate changes are disaggregated to sex- and citizenship-based cohorts, a significant heterogeneity in the effects among these cohorts is observed. For example, the unemployment rate increase in the most affected industry, *Administrative and Support Service Activities* (industry N) in year 5 is 7.22% while for various cohorts in this industry it ranges from 1.21% (for women - citizens of other countries) to 37.54% (for men - refugees). A similar pattern is observed in the second most affected industry, *Accommodation and Food Service Activities* (industry I), for which the industry-specific unemployment rate in year 5 is 6.49%, and the range across cohorts spans from 0.86% (for women - citizens of other countries) and 46.41% (for men - refugees). In the third and fourth most affected industries, *Agriculture, Forestry and Fishing* (industry A) and *Human Health and Social Work Activities* (industry Q), a similar heterogeneity is observed, however, the least affected cohort is women-natives. This disaggregated assessment is consistent with sex- and citizenship-based results presented above, which concluded that (1) natives are generally less affected than other citizen cohorts, (2) women are generally less affected than men, and (3) refugees are most strongly affected.

In sum, the model enables disaggregation of unemployment rate impacts into cohorts differentiated by sex, citizenship and industry, which allows for a systematic exploration of heterogeneous impacts of migration on the population. This provides a more comprehensive understanding of the impact of migration on the labour market and can inform policy decisions to support integration of migrants into the workforce.

## Discussion and conclusions

This paper focuses on the macroeconomic and labour market repercussions of immigration, which have a growing place in the political discourse, whether it is related to the declining labour force or the challenges related to integration in Western societies (Dustmann et al., 2017; Brell et al., 2020).

The major conclusion of this analysis is that a data-rich and large-scale macroeconomic ABM can provide detailed information on the economic impacts of a sizable migration shock and, thus, on the resilience of an economy. This has been demonstrated by our analysis of the short- to mid-term impact of migration on the labour market and the wider economy of Austria using a highly detailed ABM. For this, we augmented the ABM of Poledna et al. (2023) to include more than a thousand population cohorts, each defined by the attributes of sex, citizenship, activity status, and occupation (industry). The detailed model enabled the simulation of the labour market outcomes for each of the cohorts based on behavioural, institutional, and socio-economic factors captured by empirically estimated probabilities of employment. Considering feedback effects between the labour market outcomes and the general state of the economy and through the interaction of the agents, we simulated the effects of migration on the economy of Austria.

The overall results of the analysis corroborate previous conclusions in the literature that have shown a positive impact of refugee migration on aggregate consumption and GDP growth accompanied by a reduction of the GDP per capita (cf., e.g. Holler & Schuster, 2020). The innovation of this analysis has been to show the effects of migration differentiated across sex, citizenship, activity status, and occupation (industry). According to the macroeconomic ABM, the impact of migration on unemployment varies significantly across these cohorts. Specifically, we found that: (1) natives are generally less affected than citizens from the EU and other countries, (2) women are generally less affected than men, and (3) men from other countries are most strongly affected by migration.

The migration scenario analyzed in this paper is a hypothetical yet realistic scenario inspired by the 2015 experience in Austria. The assumption of the age and sex composition and countries of origin of the migrants are based on the actual data from that year. The migration scenario and the ABM developed for this study are specifically tailored for the small open economy of Austria, but they can be also straightforwardly adapted to different migration scenarios or larger economies, such as the US or the EU. In conclusion, our ABM is a highly detailed model that could forecast the labour market effects of future migration waves in real time and aid in informed policy-making. It highlights the importance of considering the heterogeneous impacts of migration and the need for policies that support integration and provide opportunities for newcomers to actively participate in the labour market and contribute to the economic growth of the host country.

Our approach has a number of potential limitations. First, in the model, we only consider the short-term impacts on the labour market of a large wave of migrants. We do not consider the long-term effects, which can be influenced by factors such as the return rate to the country of origin, family reunification, etc. Second, the effects are strictly measured on the labour market and do not account for other dimensions, such as the political environment that can influence the socioeconomic integration of refugees.Third, we assume that the refugees in our model have immediate access to the labour market with similar transition probabilities as other migrants from non-EU countries with similar socio-economic characteristics. However, as highlighted by Jestl et al. (2022), Bešić and Ortlieb (2023), and Jestl and Tverdostup (2023), this assumption does not fully align with the challenges faced by refugees in transitioning into the labour market and makes the migration scenario even more extreme which is useful for testing the resilience of the Austrian economy and labour market to large-scale migration shocks.

Additionally, constrained by data limitations, we do not explicitly consider the educational backgrounds and skills of individuals, which are important determinants of labour market entry and success (Jestl et al., 2022) and do not account for the regional labour market differences, which are crucial in Austria due to its regionally differentiated labour markets and relatively immobile population. These differences are particularly important in the context of filling open vacancies and the geographical distribution of job opportunities (Bacher et al., 2017; Christl, 2020). However, the labour market transition probabilities calibrated from the Register-based labour market careers data (Statistics Austria, 2022a) implicitly include these and other relevant factors. The limitations of our model underscore the need for further research and more granular data which more accurately capture differences between natives, refugees and other migrants in the labour market context (Jestl et al., 2022).

In terms of policy implications, our study shows that labour market regulation should consider the heterogeneous impacts of migration on different population groups and industries. For instance, migration shocks can lead to an increase in unemployment among refugees in certain industries, such as agriculture, hospitality, other services, and healthcare. This can be caused by a lack of qualification recognition which often drives highly qualified migrants into these sectors resulting in unemployment and low-quality jobs (Bešić & Ortlieb, 2023; Ortlieb & Weiss, 2020). One of the reasons is that the current Austrian labour market landscape typically requires formal qualifications, which migrants may not have due to differing international standards (Wittfeld, 2019). To address this issue, certification processes can be revised to reduce entry barriers for skilled migrants (including refugees) while maintaining workforce quality (Wittfeld, 2019). Additionally, private sector employers, in particular, from the potentially negatively affected industries, can be involved in providing suitable employment opportunities for migrants—which should be supported by authorities through providing more legal certainty for migrants (Torfa et al., 2023). Finally, targeted investment in the industries that may be negatively affected by migration can help to promote their growth and create additional labour demand (Fallah et al., 2019).

### Supplementary Information

**Additional file 1.** ODD+D protocol describing the ABM.

## Data Availability

Codes and calibration data of the model used for the current study are available at Zenodo, https://doi.org/10.5281/zenodo.7271552. The labour market dataset (ERV) is available from Statistik Austria, but restrictions apply to the availability of these data, which were used under license for the current study, and so are not publicly available.

## Footnotes

1 2019 is chosen as the baseline year to separate the economic impacts of the COVID-19 pandemic.

2 $2 \times 4 \times 2 \times 62 = 992$ cohorts of employed and unemployed agents; $2 \times 4 \times 1 \times 1 = 8$ cohorts of inactive agents; and 1 cohort of retired agents (sex and citizenship of retired individuals are not distinguished in the model).

3 The other possible transitions, i.e., transitions from employed to inactive as well as from unemployed to inactive and from inactive to unemployed, are omitted for simplicity.

4 We strive to model a large enough, yet still plausible, migration influx to impact the Austrian economy. For example, while the number of migrants in the migration scenario is about three times larger than the number of asylum-seekers who came to Austria in the refugee crisis of 2015 (which was above 88,000), it is close to the total of asylum applications submitted in Austria in 2022 (which was 108,780, see Bundesministerium für Inneres, 2022) combined with 87,570 temporary rights of residence issued by Austria to displaced Ukrainians in 2022 (Eurostat, 2023b). 250,000 people correspond to 2.8% of the total population in Austria in 2019 (model baseline year). For comparison, in 2022, Czechia and Poland accepted a number of refugees which is more than 3% of their population.

5 In reality, the process of the asylum procedure takes several months between the entry into the territory, the application for asylum and the decision, which, if positive, allows the refugee to enter the labour market.

6 The participation rate is the proportion of the working-age population who are in the labour market, employed or looking for work. The employment rate is the proportion of the working-age population who are employed.

7 For example, if an employee gets laid off and is replaced by a recent university graduate, the overall number of employed remains unchanged, i.e., the employment rate remains constant. However, the laid-off employee remains active in the labour market and this increases the participation rate.

8 62 industries $\times$ 2 sexes $\times$ 4 citizenship.
