# Supplementary material for: Economic and labour market impacts of migration in Austria: an agent-based modelling approach
Source: Comp Migr Stud. 2024 Mar 26;12(1):18. doi: 10.1186/s40878-024-00374-3 (PMC10965602; doi:10.1186/s40878-024-00374-3)
Supplement: Supplementary file 1 — Additional file 1. ODD+D protocol describing the ABM. [file 40878_2024_374_MOESM1_ESM.pdf]

Supplementary Information for: Economic and labour market impacts of migration in Austria: an agent-based modelling approach

1 ODD+D protocol describing the ABM

In the following, we describe the ABM following the ODD + D protocol (Müller et al., 2013).

1.1 Overview

1.1.1 Purpose

The purpose of the model is to analyze short to mid-term macroeconomic and labour market impacts of a hypothetical large-scale migration influx in Austria. In this way, the model can be useful to assess the resilience of the Austrian economy and the labour market to migration shocks. The model is designed for use by policymakers, government officials, and stakeholders in sectors affected by migration. It may also be of interest to researchers studying labour markets, migration, and its economic impacts.

1.1.2 Entities, state variables, and scales

The model consists of several types of agents: **individuals** representing the household sector, **firms**, the **general government**, the **bank**, the **central bank**, and foreign firms representing the rest of the world.

- The **individuals** are characterised by parameters[1] such as *age, sex and citizenship*. Their state variables are *activity status* (inactive, investor, worker — unemployed or employed), *occupation* (industry), *income, consumption budget, investment, capital stock* and *savings.*
- The **firms** are characterised by parameters such as *industry* and principal *product* produced, *productivity of intermediate inputs and capital*, and *capital depreciation rate.* Their state variables are *demand, supply, production, sales, inventory of finished goods, price, cost-push inflation, stock of intermediate goods/services and raw materials, capital, investment in capital stock, number of persons employed, productivity*

[1]The full list of parameters and their values is provided in Section 2.

of labour, vacancies (demand for new labour), wages paid, overall debt, profits, deposits, and *equity.*

- The **general government** is characterised by parameters such as *income tax rate*, *corporate tax rate*, *value-added tax rate*, *social insurance rate*, *export tax rate*, *tax rate on capital formation*, *tax rate on government consumption*, *unemployment benefit replacement rate.* Their state variables are *government consumption, government revenues, government deficit*, and *government debt.*

- The **bank**: is characterised by parameters such as *rate of instalment on debt*, *banks' capital ratio*, *loan-to-value (LTV) ratio.* Their state variables are *equity, loans extended, deposits held, profits* and *reserves/advances from/to the central bank.*

- The **central bank**: is characterised by parameters such as *inflation target of the monetary authority*, *real equilibrium interest rate*, *adjustment coefficient of the policy rate*, *weight of the inflation target*, *weight of economic growth.* Their state variables are *policy rate, profits, equity, government bonds* and *net credit/debt to the rest of the world.*

The main exogenous driver of the model is a migration influx from abroad. The model can be also affected by export demand and import supply constraints as well as by changes in government consumption.

The model operates on a national scale representing the Austrian economy as a whole without differentiating between regions or locations within Austria. Therefore, the model does not explicitly incorporate a spatial dimension. The model is simulated over a fixed 5-year period. The temporal resolution of the model (simulation time step) is one-quarter.

1.1.3 Process overview and scheduling

During each time step of the model simulation, a series of activities occur in the following order sequence:

1. Individuals migrate to the model environment (if applicable to the given time step)

2. Firms

   (a) Firms form expectations for economic growth and inflation

   (b) Firms set prices and determine supply based on the expectations

(c) Firms obtain external financial resources (loans from the bank)
(d) Firms adjust their employment through hiring/firing
(e) Firms produce based on available labour, intermediate goods/services and capital
(f) Firms invest in their capital stock
(g) Firms buy intermediate goods and services to adjust their inventory
(h) Firms account for their profits, deposits and the overall debt
(i) Firms may go bankrupt

3. Individuals
   (a) Individuals interact with firms on the labour market
   (b) Individuals determine consumption based on the expected income
   (c) Individuals buy consumption goods
   (d) Individuals invest in their capital stock (e.g., housing)
   (e) Individuals receive income
   (f) Individuals make savings

4. The government
   (a) The government consumes
   (b) The government determines its revenue (i.e., from taxes)
   (c) The government estimates its deficit
   (d) The government estimates its debt

5. The bank
   (a) The bank provides loans to firms
   (b) The bank accounts for profits and losses

6. The central bank
   (a) The central bank determines the policy rate
   (b) The central bank accounts for profits and losses

The order of agent activations is randomized each time step.

1.2 Design concepts

1.2.1 Theoretical and empirical background

The design of the model at the system level reflects the complexity inherent in economic systems, where multiple agents interact in interconnected ways. It is underpinned by several concepts:

- Agent heterogeneity: The model is populated by heterogeneous agents who represent natural persons (individuals) or legal entities (firms). These agents are not uniform in their preferences and capabilities, reflecting the diversity found in real-world economic actors.

- Emergence: Macroeconomic dynamics emerge bottom-up from micro-level interactions rather than being imposed top-down. Aggregate model behaviour arises from interactions of heterogeneous agents.

- Data richness: The model is built around publicly available macro- and microdata. The model incorporates all economic activities, as classified by the European System of Accounts (ESA).

To more accurately reflect real-world behaviours and interactions in economic systems, the agents' decision models are based on two key assumptions:

- Bounded rationality: Agents do not fully understand the complex structure of their economic environment and use simple heuristics for decision-making, reflecting a departure from the rational expectations hypothesis.

- Adaptive expectations and learning: Agents use simple AR(1) rules to forecast key variables (i.e., expected growth rate and expected inflation) based on past realizations. They continuously learn and update the parameters of these forecasting rules.

The use of heuristics and adaptive expectations follows related literature on behavioural models in macroeconomics and agent-based modelling (Dawid and Delli Gatti, 2018). The forecasting rules were chosen as the most parsimonious yet empirically relevant option.

The model is grounded in empirical data derived from various sources, such as national accounts, sector accounts, input-output tables, business demography data, population census, labour market career statistics, government statistics, Basel III, ECB statutes, banking practices, and literature.

These data were available at multiple levels of aggregation, ranging from the micro-level (e.g., business demography and census data) to the macro-level (e.g., national and sector accounts, input-output tables).

1.2.2 Individual decision making

The subjects of decision-making in the model are various types of agents, i.e., individuals, firms, the bank, the central bank, and the government. Agents of each type make decisions based on their specific context: individuals make decisions about labour supply, consumption, and savings, firms make decisions about pricing, production, investment, financing, and employment, the bank makes decisions about lending and interest rates, the central bank makes decisions about monetary policy and the government — about the fiscal policy. Decision-making is modelled at the individual agent level. There is no group-level decision-making.

The agents' decision-making is based on bounded rationality, where agents make decisions with limited information. They rely on heuristics, rules of thumb, and simple forecasting models to make decisions under uncertainty. For example, individuals follow a rule of thumb to consume a fraction of their expected income. Firms use simple adaptive rules to forecast demand. They do not optimize any explicit objectives or have well-defined utility functions. There are no explicit success criteria for economic optimization. However, agents update expected values based on past errors, so they could be assumed to try to improve individual forecasts.

Agents make decisions based on a combination of heuristics, past experiences, and current economic conditions. For example, individuals plan consumption expenditure as a fraction of disposable income expectations and allocate consumption to different goods using fixed coefficients; firms set prices and production quantities based on adaptive expectations of economic growth and inflation, estimate future demand, costs, and profits using simple AR(1) forecasting rules and make investment, employment, and financing decisions based on these forecasts; the bank lends to firms subject to risk constraints and capital requirements and sets lending rates as a markup on central bank policy rate; the central bank sets policy rate according to a Taylor-type rule responding to growth and inflation; the government determines fiscal policy (spending, taxes, social transfers) based on historical data.

The agents adapt their behaviour to endogenous and exogenous state variables. For example, firms form expectations for demand and price of their product based on the demand and price of the previous time step

(endogenous variables) and expected overall economic growth and inflation (exogenous variables). Individuals form expectations for their disposable net income based on their activity status (endogenous variable) as well as the consumer price index of the previous time step and expectations of the inflation rate (exogenous variables). As the agents face fundamental or "Knightian" uncertainty about economic structures and future developments, forecasting and expectation errors are persistent due to structural changes and exogenous shocks.

The future-orientation of forecasting and decisions as well as backward-looking learning dynamics introduce intertemporal considerations in the model. In the long run, adaptive learning over time leads to convergence to an equilibrium where agents use optimal linear forecasts. Furthermore, the model incorporates a time-consuming capital adjustment process. New capital goods bought at a certain time will only be part of the capital stock in the next period, making capital a durable and sticky input.

Social norms and cultural values play an implicit role in the decision-making processes of individuals concerning changes in their activity status, as labour market hazard rates used to calibrate these decisions include these factors as well (Brügger et al., 2009)[2].

Spatial aspects do not play a role in the decision-making process of agents.

1.2.3 Learning

The decision-making process of agents incorporates (collective) learning, allowing them to adapt their decision rules over time based on their experiences and expectations. Agents form expectations about key variables like economic growth and inflation using simple autoregressive forecasting models. They estimate the parameters of these parsimonious models based on limited historical information. Over time, they update the estimated parameters as new data becomes available and forecast errors arise. Thus, agents collectively learn the optimal parameters to approximate the unknown true economic dynamics. In the long run, unless disturbed by exogenous shocks or endogenous fluctuations, learning leads the model economy to converge to an equilibrium where agents use the optimal univariate linear forecasting rules.

[2] For more details on the labour market hazard rates, see subsection 1.3.4.

1.2.4 Individual sensing

In their decision-making process, agents sense and consider both endogenous and exogenous state variables. For instance, firms sense endogenous variables such as their own past production, sales, and prices and exogenous variables such as overall economic growth and inflation rate, government spending and Euro area growth and inflation.

The sensing process can be erroneous since agents have limited information and face fundamental uncertainty (see subsection 1.2.2) and thus form expectations about the future that may not correspond to actual realizations. For example, the difference between a firm's production and sales is a reflection of the firm's expectation error concerning demand.

Agents can perceive certain state variables of other agents which influence their decision-making processes. For example, individuals perceive the prices of goods and services offered by firms and make consumption decisions based on these prices; firms assess potential customers and business partners through the search and matching mechanism when trading and select the individuals to hire based on their attributes such as sex, citizenship, activity status, and previous employment industry; the bank gauges the financial positions of firms when deciding on lending.

Agents have local perceptions from matching on goods and labour markets, plus visibility into national aggregates such as inflation and economic growth. Overall, the agents are assumed to have limited information and face fundamental uncertainty about the economy's dynamics. Information limitations are captured through bounded observation rather than access constraints.

There are no costs for cognition and costs for gathering information included in the model.

1.2.5 Individual prediction

Individuals and firms collectively estimate future output and inflation using adaptive/learning forecasting models (AR(1)). These models are lean univariate linear models with only two estimated parameters and are estimated using historical time series data on aggregate output and inflation. Based on these expectations as well as demand and prices on their production on the previous time step, firms set prices and determine supply.

The bank assesses borrowing firm risks based on their leverage ratios. Budgeting and policy decisions of the government (except for government consumption of goods and services) and the central bank are not based

on forward-looking internal forecasting models, but rather on an exogenous process that determines fiscal and monetary policies.

Agents can be erroneous in their predictions. For instance, firms face fundamental uncertainty regarding future sales, market prices, and the availability of inputs for the production process, among others. Consequently, each firm has to form expectations about the future that may not correspond to actual realizations. For example, a firm's sales are the realized demand dependent on the supply available from the firm after the production process has taken place. The difference between production and sales is excess supply, which is a reflection of firms' expectation error concerning demand. This is implemented computationally by stochastic shocks to the AR(1) forecasting models and reestimation of AR parameters to adapt expectations on each time step.

1.2.6 Interaction

There are both direct and indirect interactions among agents, primarily through market mechanisms and search-and-matching processes in the labour and goods/services markets. These decentralized markets directly connect counterparties for trade. The matching algorithms provide explicit contractual relationships. Firms and banks also directly interact for lending and interest payments. The government collects taxes and distributes transfers. These interactions have immediate transactional impacts locally but also diffuse systemic consequences.

Interactions between agents depend on decentralized matching in markets based on price signals and random search, financial relationships for lending and interest charges contingent on leverage conditions and tax payments and government transfers depending on income and activity status. For example, individuals actively search for the best bargain on the goods market, i.e., the lowest price, to satisfy their consumption demand for products. They visit a number of randomly chosen firms to fulfil their needs. The probability of a firm being chosen by a consumer is influenced by the price charged by the firm and the relative size of the firm compared to other firms. Firms charging a lower price and larger firms have a higher probability of being picked by consumers.

Communication among agents is mediated entirely through revealed economic actions and quantities and is not a direct form of communication like messaging or negotiation but rather an abstract representation of market interactions.

There are no coordination networks between the agents.

1.2.7 Collectives

The agents do not explicitly form organizations or coordinated groups. However, each firm is part of an industry with a number of firms in each industry. The industries correspond to the NACE 2-digit classification. Individuals are grouped into cohorts of the same sex, citizenship, activity status and industry of occupation (e.g., a cohort of women natives employed in the agricultural industry). These aggregates are statistical composites rather than distinct decision-making collectives.

Firms and individuals are modelled as individual agents with their own state variables and traits. These agents interact with each other, and in this way, the collective behaviour of the economy emerges from these interactions rather than being explicitly defined. On the other hand, the bank is a singular/representative bank that pools lending and deposits across other institutional sectors, the central bank is a singular entity responsible for the monetary policy, and the government is an exogenous collective actor that makes fiscal policy choices (except for government consumption of goods and services which is attributed to individual government entities).

1.2.8 Heterogeneity

The agents are heterogeneous. For example, individuals vary by activity status and income sources, firms differ in size, industry, production technology parameters, and wages. Decision variables like the consumption composition of individuals and output and pricing strategies of firms are agent-specific. All agents have separate balance sheets depicting assets, liabilities, and ownership structures. There is differentiation both by institutional domain and within industries based on state variables like technology or employment. Firms in the same industry can still make disjoint decisions.

Firms and individuals use similar forecasting heuristics (i.e., the used AR(1) prediction model is homogeneous across all agents) but apply them to different decision objects (output/pricing vs consumption/savings).

1.2.9 Stochasticity

Randomness plays a key role in representing uncertainty, matching frictions, and structural instability. It is embedded in market interactions, expectations, and external drivers. In particular, the following processes involve randomness or stochastic elements:

- Initialization: Firm sizes are randomly drawn from a power law distributions

- Expectation formation: Random shocks are introduced to output and inflation forecasting models
- Search and matching in decentralized markets: Random matching of buyers and sellers as well as employers and job seekers. Probabilities of matching occurrence depend partly on a random search (e.g., if there are several firms of the same size selling a good for the same price, a consumer will choose one of them randomly or if there are several potential employees with the same properties, a firm will choose one of them randomly).
- Exogenous shocks: Government consumption, imports and exports include stochastic elements. Stochastic shocks create realistic disequilibrium dynamics rather than steady optimal growth.

Since stochastic shocks lead to some randomness across runs even when starting from the same reference point, Monte Carlo methods are used to run an ensemble of simulations.

1.2.10 Observation

Macroeconomic variables such as GDP, GDP per capita, and government debt, labour market aggregates such as unemployment, employment and participation rates as well as disaggregated unemployment (in absolute terms) and unemployment rates by population cohort are collected on each simulation time step.

The following key macroscopic results and characteristics emerge from interactions of the individual agents: aggregate output (GDP) emerges from decentralized production, pricing and trade decisions; economy-wide inflation rate emerges from individual price-setting behaviours; tax revenues are an aggregate from heterogeneous income sources; consumption and investment reflect aggregate household budgets; unemployment rates emerge from hiring and dismissal decisions of firms and changes of activity statuses of individuals.

1.3 Details

1.3.1 Implementation Details

The model was implemented in MATLAB software. The results of the simulations were analysed and visualized in MATLAB and Excel. The migration

scenarios and the data on labour market transition probabilities were prepared using Python. The source code of the model is available from the authors upon request.

1.3.2 Initialization

The initial conditions of the model are set to represent the Austrian economic and demographic structure for the fourth quarter of 2019.

Initial attributes of individuals, firms, the bank, the central bank and the government are set based on empirical data. These include income, balance sheet positions, and capital stocks. Economy-wide positions are calibrated using national and sectoral account statistics, e.g., total deposits, government debt, and household capital. Macroeconomic aggregates match historical data, individual firm sizes are skewed using distributions and the number and properties of individuals match the population census.

The initialisation of the model is always the same, except for the size of the firms which are drawn from a power law distribution.

1.3.3 Input data

The model uses the following external data sources:

- The initial size of all cohorts of individuals is calibrated based on the *register-based census (Registerzählung)* (Statistics Austria, 2022b).
- Labor market transition probabilities are estimated from the *register-based labour market careers data* (Statistics Austria, 2022a)
- Migration arrival pattern is based on the 2015 *asylum applications* data (Eurostat, 2023) and *Displaced Persons in Austria Survey (DiPAS)* conducted by the Wittgenstein Centre for Demography and Global Human Capital (Buber-Ennser et al., 2016).
- The distribution of refugees across industries is based on the data retrieved from the online Database of Labour Market Information (*Arbeitsmarktinformationssystem, amis*) maintained by the Austrian Federal Ministry of Labour and Economy (Bundesministerium für Arbeit und Wirtschaft, 2023).
- Initial values of various agents' state variables are calibrated from Eurostat data tables enlisted in Table 1.

Table 1: Eurostat data tables

Name	Code
Business demography by legal form (from 2004 onwards, NACE Rev. 2)	bd_9ac_l_form_r2
Symmetric input-output table at basic prices (product by product)	naio_10_cp1700
Cross-classification of fixed assets by industry and by asset (stocks)	nama_10_nfa_st
Government revenue, expenditure and main aggregates	gov_10a_main
General government expenditure by function (COFOG)	gov_10a_exp
Quarterly non-financial accounts for general government	gov_10q_ggnfa
Quarterly government debt	gov_10q_ggdebt
Financial balance sheets	nasq_10_f_bs
Non-financial transactions (annually)	nasa_10_nf_tr
Non-financial transactions (quarterly)	nasq_10_nf_tr
GDP and main components (output, expenditure and income)	namq_10_gdp
Money market interest rates - quarterly data	irt_st_q

Note: The codes under which the respective datasets are available from Eurostat (such as, e.g. naio_10_cp1700) are shown in the second column.

1.3.4 Submodels

- **Labor market submodel**:

  The labour market submodel is a development of the corresponding module of the model by Poledna et al. (2023). Compared to the original version, it takes into account the heterogeneity of individuals concerning sex, citizenship, activity status, and industry of occupation, which is reflected in distinct probabilities to be hired by firms from various sectors. This submodel captures labour demand and supply, transitions between activity statuses of individuals and probabilistic matching on the labour market grounded in empirical data (Statistics Austria, 2022a).

  - **Entities and scales**: 1001[3] individual cohorts representing persons, differentiated by sex, citizenship (native, EU, other, refugee), activity status (employed, unemployed, inactive), and industry of occupation (62 industries according to NACE classification). Retired individuals exist as a separate cohort which does not participate in the labour market, but receives social benefits from the government
  - **Decision-making**: Firms determine labour demand (i.e., target employment level) based on expectations of economic activity

[3] $2 \times 4 \times 2 \times 62 = 992$ cohorts of employed and unemployed agents; $2 \times 4 \times 1 \times 1 = 8$ cohorts of inactive agents; and 1 cohort of retired agents (sex and citizenship of retired individuals are not distinguished in the model).

and average labour productivity. They post vacancies or dismiss staff based on the difference between the desired and existing workforce.

– **Interactions**: Vacancies are filled through a search and matching process between firms and job seekers. Transition probabilities govern hiring likelihood for individuals from each cohort based on empirical job mobility data (Statistics Austria, 2022a). Compared to the original version of the labour market module in Poledna et al. (2023), all agents of working age can be hired, however, the probability of being hired depends on their activity status, along with their sex, citizenship and industry of occupation. Namely, new hires are determined by the transition matrix P_{gx} where $x = 1, \ldots, X$ is the cohort id ($X = 1000$ is all possible combinations of sex, activity status, citizenship and industry of occupation (in case of unemployed individuals, the industry of last occupation before unemployment) and $g = 1, \ldots, G$ is the industry, to which the hiring firm belongs ($G = 62$ is the number of industries according to NACE-level classification). Therefore, P_{gx} is a 62×1000 matrix that specifies the hazard rates for individuals from each cohort to be hired by a firm from a particular industry. Matches are made based on firms' open vacancies and applicants from all cohorts according to the labour market transition probabilities. The concrete individuals from the identified cohort are selected randomly. The iterative process continues until each vacancy is filled. Wages paid vary by industry and employment status (overtime/undertime vs full-time). Separation can occur due to layoffs (i.e., target employment of a firm is lower than the current employment) or voluntary transitions between firms. The individuals to be dismissed are selected randomly.

– **Initialisation**: The initial number of individuals in each cohort in each cohort (except for cohorts of refugees) is based on the register-based census (*Registerzählung*) as of 31 October 2018, see Statistics Austria (2022b). These data reflect all persons residing in Austria on a given date and their various attributes, including, sex, activity status, citizenship and industry of occupation. The sizes of the refugee cohorts are estimated based on the data from UNHCR (UNHCR, 2023) and Online Database of Labour Market Information (Bundesministerium für Arbeit und

Wirtschaft, 2023).

The initial employment of firms is initialized by sampling from power law firm size distribution based on business demography statistics. Initial wages for each cohort are calibrated based on input-output tables and census employment data. Firm populations for each industry are kept constant but initial employment of each firm is drawn from the firm size distribution per industry.

– **Emergence**: Unemployment rates for each cohort arise from decentralized hiring and separation decisions. The aggregate unemployment is an emergent property from micro-level labour market dynamics.

2 Model parameters

The classification of the model parameters according to their data source and calibration method is shown in Table 2.

Table 2: Model parameters

Parameter	Description	Value	Source
G/S	Number of products/industries	62	census data, business demography data
H^{act}	Number of economically active persons	5584893	
H^{inact}	Number of economically inactive persons	1576608	
J	Number of government entities	42917	
L	Number of foreign consumers	85835	
I_s	Number of firms/investors in the sth industry	see Table 3	
$\bar{\alpha}_i$	Average productivity of labour of the ith firm	parameters are firm/sector specific; see Table 3	input-output tables
κ_i	Productivity of capital of the ith firm		
β_i	Productivity of intermediate consumption of the ith firm		
δ_i	Depreciation rate for capital of the ith firm		
$\bar{w}_i$	Average wage rate of firm i		
a_{sg}	Technology coefficient of the gth product in the sth industry		
b_g^{CF}	Capital formation coefficient of the gth product (firm investment)		
b_g^{CFH}	Household investment coefficient of the gth product		
b_g^{HH}	Consumption coefficient of the gth product of households		
c_g^{G}	Consumption of the gth product of the government in mln. Euro		
c_g^{E}	Exports of the gth product in mln. Euro		
c_g^{I}	Imports of the gth product in mln. Euro		
τ_i^{Y}	Net tax rate on products of the ith firm		
τ_i^{K}	Net tax rate on production of the ith firm		
τ^{INC}	Income tax rate	0.2177	government statistics, sector accounts
τ^{FIRM}	Corporate tax rate	0.1027	
τ^{VAT}	Value-added tax rate	0.1489	
τ^{SIF}	Social insurance rate (employers' contributions)	0.2136	
τ^{SIW}	Social insurance rate (employees' contributions)	0.1722	
τ^{EXPORT}	Export tax rate	0.0023	
τ^{CF}	Tax rate on capital formation	0.1068	
τ^{G}	Tax rate on government consumption	0.0102	
r^{G}	Interest rate on government bonds	0.0052	
μ	Risk premium on policy rate	0.0118	
ψ	Fraction of income devoted to consumption	0.981	
ψ^{H}	Fraction of income devoted to investment in housing	0.0769	
θ^{DIV}	Dividend payout ratio	0.7438	
θ^{UB}	Unemployment benefit replacement rate	0.3562	Basel III, ECB statutes, banking practices, literature
θ	Rate of instalment on debt	0.05	
ζ	Banks' capital ratio	0.03	
ζ^{LTV}	Loan-to-value (LTV) ratio	0.6	
ζ^{b}	Loan-to-capital ratio for new firms after bankruptcy	0.5	
π^*	Inflation target of the monetary authority	0.005	
α^{G}	Autoregressive coefficient for government consumption	0.9872	national accounts (exogenously estimated)
β^{G}	Scalar constant for government consumption	0.1286	
σ^{G}	Standard deviation of government consumption	0.0078	
α^{E}	Autoregressive coefficient for exports	0.9788	
β^{E}	Scalar constant for exports	0.2313	
α^{I}	Autoregressive coefficient for imports	0.9819	
β^{I}	Scalar constant for imports	0.1974	
$\alpha^{\text{Y}^{\text{EA}}}$	Autoregressive coefficient for euro area GDP	0.9829	
$\beta^{\text{Y}^{\text{EA}}}$	Scalar constant for euro area GDP	0.2538	
$\alpha^{\pi^{\text{EA}}}$	Autoregressive coefficient for euro area inflation	0.3712	
$\beta^{\pi^{\text{EA}}}$	Scalar constant for euro area inflation	0.0024	
$\sigma^{\pi^{\text{EA}}}$	Standard deviation of euro area inflation	0.0022	
ρ	Adjustment coefficient of the policy rate	0.9575	
r^*	Real equilibrium interest rate	-0.008	
ξ^{π}	Weight of the inflation target	0.6081	
ξ^{γ}	Weight of economic growth	1.8663	
C	Covariance matrix of euro area GDP and imports and exports		

Note: Model parameters for the reference quarter 2019:Q4. Exogenous autoregressive coefficients and parameters of the Taylor rule are estimated over the sample 1997:Q1 to 2019:Q4.

Table 3: Sectoral parameters

	I_s	N_s	α_s	β_s	κ_s	δ_s	w_s	τ_s^{Y}	τ_s^{K}	b_g^{CF}	b_g^{CFH}	b_g^{HH}	c_g^{G}	c_g^{E}	c_g^{I}
A01	4000	13042	0.1278	1.6429	0.0459	0.0127	0.0049	0.0107	-0.2817	0.0017	0.0006	0.0111	0	0.0051	0.0172
A02	200	6788	0.0946	2.0298	0.2081	0.014	0.0087	0.0135	-0.0243	0	0	0.0024	0	0.0005	0.0039
A03	20	238	0.0984	1.5345	0.051	0.0088	0.0045	0.0264	0.0034	0	0	0.0005	0	0	0.0006
B	253	5904	0.0851	1.7856	0.1809	0.0365	0.0124	0.0071	-0.001	0.0003	0.008	0.0002	0	0.0027	0.0317
C10-12	1781	77053	0.0762	1.3742	0.5738	0.0275	0.0083	0.004	-0.0062	0	0	0.0626	0	0.0614	0.0539
C13-15	470	14259	0.0551	1.4885	0.3705	0.0216	0.0098	0.0044	-0.0043	0.003	0	0.0324	0	0.0216	0.0522
C16	934	30148	0.0746	1.3614	0.5224	0.0202	0.0088	0.003	0.0049	0.0025	0.0662	0.0015	0	0.0219	0.0103
C17	465	16157	0.1061	1.451	0.4511	0.026	0.0137	0.0024	-0.0012	0	0	0.0018	0	0.025	0.0163
C18	160	9937	0.0593	1.6476	0.3126	0.0302	0.0121	0.0022	0.0048	0	0	0	0	0.0015	0.0006
C19	7	1380	0.5714	1.1285	0.6619	0.027	0.0138	0.0061	-0.0019	0	0	0.0086	0	0.0075	0.0219
C20	296	18290	0.2021	1.2948	0.7194	0.0257	0.0123	0.0025	0.0017	0	0.0025	0.0078	0	0.07	0.0786
C21	65	15359	0.0533	1.7314	0.1758	0.0148	0.0095	0.0026	0.011	0	0	0.0056	0.0232	0.0211	0.0312
C22	478	29708	0.0526	1.5909	0.3912	0.0303	0.0099	0.0027	0.0106	0.0025	0.0121	0.0033	0	0.0255	0.0301
C23	709	29736	0.0511	1.611	0.2613	0.0241	0.0105	0.0059	0.0103	0.0023	0.0308	0.0014	0	0.0124	0.0137
C24	1114	37639	0.107	1.3363	0.5202	0.0303	0.0124	0.0041	0.0084	0.0054	0	0	0	0.0562	0.0461
C25	1007	74879	0.0486	1.6195	0.4459	0.0252	0.01	0.002	0.0059	0.0136	0.0122	0.002	0	0.0417	0.0386
C26	339	22661	0.0806	1.6594	0.2823	0.0396	0.0108	0.0022	0.003	0.0416	0	0.0096	0.0006	0.0408	0.0557
C27	462	44524	0.0558	1.7342	0.3037	0.04	0.0096	0.0014	0.0023	0.0291	0.0014	0.0057	0	0.0501	0.0448
C28	1069	82905	0.0665	1.4894	0.5563	0.0365	0.0109	0.0016	0.0094	0.1127	0.0017	0.0015	0	0.1054	0.094
C29	190	37281	0.1028	1.2792	0.4357	0.0328	0.0073	0.0018	0.0029	0.0713	0	0.0169	0	0.0848	0.0878
C30	60	11601	0.1053	1.4041	0.9409	0.0612	0.0138	0.002	0.0081	0.0206	0	0.0022	0.0002	0.022	0.015
C31_32	1584	39488	0.0444	1.6065	0.4489	0.0228	0.0083	0.0062	0.0126	0.0312	0	0.0197	0.005	0.0219	0.0282
C33	841	22716	0.0971	1.7404	1.522	0.0009	0.0238	0.0032	0.0152	0.0371	0	0	0	0.0076	0.009
D	2403	5151	1.3825	1.2704	0.26	0.0216	0.0926	0.0013	0.0093	0	0	0.0254	0	0.0072	0.0067
E36	350	752	0.3819	2.4339	0.053	0.0105	0.0451	0.0036	0.0231	0	0	0	0	0	0
E37-39	2273	14341	0.1301	1.7588	0.083	0.0134	0.0156	0.0134	0.0087	0	0	0	0	0.0046	0.0111
F	16650	270639	0.0537	1.6311	0.4983	0.0155	0.0098	0.0052	0.0112	0.2599	0.7077	0.0044	0	0.0043	0.0023
G45	4141	73031	0.034	2.002	0.4775	0.0127	0.0089	0.0054	0.0193	0.0158	0	0.026	0	0.0055	0.0003
G46	15937	187241	0.0518	2.354	0.4454	0.0244	0.0126	0.0056	0.0143	0.0391	0.017	0.0392	0.0101	0.0648	0.0051
G47	14147	330416	0.0187	2.6239	0.3353	0.0161	0.0063	0.0089	0.0221	0.0067	0.0357	0.115	0.008	0.001	0
H49	4633	97230	0.0457	2.2617	0.1738	0.0176	0.0107	0.0242	0.0205	0.0017	0.0025	0.0269	0.0275	0.0392	0.0362
H50	74	293	0.1202	1.3328	0.3334	0.0309	0.0076	0	0.0035	0	0	0.0004	0	0.0021	0.0073
H51	144	8584	0.0924	1.3013	0.5643	0.0544	0.0131	0.0012	0.0101	0	0	0.0086	0	0.0041	0.0048
H52	1209	52277	0.0556	2.5981	0.0548	0.0125	0.0121	0.0053	0.0195	0.001	0.0008	0.0056	0.0245	0.0187	0.0153
H53	127	23749	0.0334	1.9449	0.9639	0.0292	0.01	0.0101	0.0214	0	0	0.0015	0	0.0037	0.002
I	15092	208578	0.0362	2.7837	0.2914	0.0102	0.0086	0.0156	0.0085	0	0	0.1302	0.0001	0.0146	0.011
J58	917	13891	0.0615	1.632	1.3036	0.0486	0.0133	0.0012	0.0045	0.0063	0	0.0083	0.0008	0.0049	0.0093
J59_60	1014	11728	0.0672	1.6064	0.6169	0.0548	0.0122	0.0016	0.0006	0.0024	0	0.0029	0.0096	0.0023	0.0062
J61	279	15176	0.1145	1.8783	0.1352	0.0349	0.0149	0.0023	0.0108	0	0	0.014	0	0.0043	0.0034
J62_63	6764	66316	0.0686	2.0502	1.1488	0.0916	0.0181	0.0027	0.0183	0.105	0	0.0002	0	0.0254	0.0182
K64	818	74191	0.0563	2.4177	0.2715	0.0299	0.0172	0.0448	0.0975	0	0	0.013	0.0005	0.013	0.0103
K65	103	27137	0.0697	1.885	0.5564	0.0209	0.0174	0.0478	0.022	0	0	0.0262	0	0.0046	0.0041
K66	2463	13134	0.0737	1.6453	2.4855	0.0473	0.0115	0.0286	0.0116	0	0	0.0018	0	0.0008	0.0004
L68A	13447	46035	0.3046	3.1554	0.0831	0.0252	0.0107	0.0143	0.0104	0.0068	0.0164	0.1874	0.0007	0.0008	0.0007
M69_70	11162	96577	0.0515	2.1928	0.8373	0.0103	0.0143	0.0041	0.0185	0.0008	0.0021	0.0027	0.0001	0.0168	0.0149
M71	6143	52187	0.0512	2.3856	0.4722	0.0324	0.0124	0.003	0.0171	0.0258	0.0771	0	0.0016	0.013	0.0083
M72	884	15279	0.2166	4.1809	1.2471	0.1726	0.0746	0.0119	0.0107	0.1493	0	0	0.0042	0.0123	0.0037
M73	3621	23904	0.0702	1.5457	1.7255	0.0399	0.0087	0.0102	0.0083	0	0	0	0.0001	0.0074	0.015
M74_75	1936	10023	0.0614	2.0119	0.8185	0.0198	0.0072	0.0071	0.0043	0	0	0.0021	0.0002	0.0014	0.0009
N77	1643	9897	0.2401	3.302	0.0943	0.0726	0.0121	0.006	0.0047	0	0	0.0076	0.0013	0.0071	0.007
N78	995	88010	0.0165	8.7166	5.5924	0.0281	0.0107	0.0009	0.0478	0	0	0	0	0.0011	0.0029
N79	1266	12010	0.0569	1.2776	1.1387	0.028	0.0093	0.0088	0.0099	0	0	0.0122	0.0015	0.0003	0.0004
N80-82	3482	113338	0.0215	2.8954	0.7472	0.0207	0.0073	0.0058	0.0252	0.0017	0.0051	0.0065	0.0072	0.0019	0.0028
O	10000	648776	0.0099	3.4772	0.1168	0.0127	0.0044	0.0482	0.0188	0	0	0.0005	0.3116	0.0011	0.0004
P	2028	167236	0.031	6.9681	0.1621	0.0191	0.018	0.032	0.0257	0	0	0.0186	0.2118	0.0002	0.0004
Q86	1594	127148	0.0532	3.599	0.207	0.0138	0.0195	0.0511	0.0125	0	0	0.0366	0.2464	0.0016	0.0012
Q87_88	805	145510	0.016	3.4278	0.2498	0.0105	0.0082	0.0333	-0.0358	0	0	0.0254	0.0628	0	0.0023
R90-92	1542	18304	0.0568	3.4079	0.2401	0.0262	0.015	0.0233	-0.0086	0.0025	0	0.0131	0.0126	0.0027	0.0025
R93	2011	17726	0.0356	2.5057	0.0816	0.0095	0.0075	0.0193	0.004	0	0	0.0104	0.0046	0.0003	0.0002
S94	1076	51845	0.0195	2.7551	0.1635	0.0084	0.0087	0.0706	0.0283	0	0	0.0106	0.0219	0	0
S95	246	2265	0.1396	2.7296	2.5608	0.2387	0.0271	0.0057	0.0124	0	0	0.0018	0	0.0001	0.0002
S96	1746	39255	0.0252	3.5302	0.1649	0.0131	0.006	0.0103	0.015	0	0	0.0179	0.0017	0	0.0004

Note: Sectoral parameters are shown for 2019:Q4.

References

B. Brügger, R. Lalive, and J. Zweimüller. Does Culture Affect Unemployment? Evidence from the Röstigraben. Discussion Paper 4283, IZA, Bonn, Germany, 2009. URL https://docs.iza.org/dp4283.pdf.

I. Buber-Ennser, J. Kohlenberger, B. Rengs, Z. Al Zalak, A. Goujon, E. Striessnig, M. Potančoková, R. Gisser, M. R. Testa, and W. Lutz. Human Capital, Values, and Attitudes of Persons Seeking Refuge in Austria in 2015. *PLOS one*, 11(9):e0163481, 2016. doi: 10.1371/journal.pone.0163481.

Bundesministerium für Arbeit und Wirtschaft. Online database - Labour market information, 2023. URL https://www.dnet.at/Amis/Datenbank/DB_IndexEN.aspx.

H. Dawid and D. Delli Gatti. *Chapter 2 - Agent-Based Macroeconomics*, volume 4 of *Handbook of Computational Economics*, pages 63–156. Elsevier, 2018. doi: 10.1016/bs.hescom.2018.02.006.

Eurostat. Asylum applicants by type of applicant, citizenship, age and sex - monthly data [migr_asyappctzm], 2023. URL https://ec.europa.eu/eurostat/web/products-datasets/-/migr_asyappctzm.

B. Müller, F. Bohn, G. Dreßler, J. Groeneveld, C. Klassert, R. Martin, M. Schlüter, J. Schulze, H. Weise, and N. Schwarz. Describing human decisions in agent-based models – ODD+D, an extension of the ODD protocol. *Environmental Modelling & Software*, 48:37–48, 2013. doi: 10.1016/j.envsoft.2013.06.003.

S. Poledna, M. G. Miess, C. Hommes, and K. Rabitsch. Economic forecasting with an agent-based model. *European Economic Review*, 151:104306, 2023. doi: 10.1016/j.euroecorev.2022.104306.

Statistics Austria. Register-based labour market careers, 2022a. URL https://www.statistik.at/en/statistics/labour-market/employment/labour-market-careers/register-based-labour-market-careers.

Statistics Austria. Population census, 2022b. URL https://www.statistik.at/en/about-us/surveys/register-based-census/population-census.

UNHCR. Refugee data finder, 2023. URL https://www.unhcr.org/refugee-statistics/download/?url=QLgW3P.
